# Respiratory syncytial virus positivity among hospital admissions for acute respiratory illness in children younger than 5 years of age in low- and middle-income countries: a systematic review and meta-analysis

**DOI:** 10.1186/s12889-026-26743-4

**Published:** 2026-02-24

**Authors:** Chelsea S. Lutz, Haijun Zhang, Maria Deloria Knoll, Erin G. Sparrow, Huiyao Chen, Daniel R. Feikin

**Affiliations:** 1 Department of International Health, Bloomberg School of Public Health, Johns Hopkins University, Baltimore, MD USA; 2 School of Public Health, Tongji Medical College, Huazhong University of Science and Technology, No.13, Hangkong Road, Wuhan, 430030 China; 3 Department of Immunization, Vaccines and Biologicals, World Health Organization, Geneva, Switzerland

**Keywords:** Acute respiratory infections, ARI, Children, Hospitalization, Low- and middle-income countries, Respiratory syncytial virus, RSV

## Abstract

**Objectives:**

To estimate the proportion RSV-positive among children aged < 5 years hospitalized with ARI in low- and middle-income countries (LMIC), where 97% of RSV mortality occurs.

**Methods:**

We conducted a systematic literature search for studies conducted pre-COVID-19 and published 2010—2022 (PROSPERO registration CRD42022361351). We estimated the RSV percent positivity and 95% confidence interval (CI) using random-effects meta-analyses. We assessed heterogeneity in RSV percent positivity using subgroup analyses and univariable meta-regression models. We assessed the influence of study sample size in sensitivity analyses.

**Results:**

Seventy-three studies conducted in 37 LMICs were included. The summary estimate of percent RSV-positive from the meta-analysis of children < 5 years hospitalized with ARI was 26.2% (95% CI: 24.3–28.3%), ranging from 18.9% (16.4–21.6%) among children 6– < 60 months to 41.3% (36.4–46.4%) among children 0– < 6 months. Only five studies included children aged < 2 months, but RSV positivity was high among this group (40.2% [35.8–44.7%]). Percent positivity stratified by WHO region ranged from 23.6% in the Africa and Southeast Asian regions to 37.5% in the European region. RSV positivity was similar across country income groups. Univariable meta-regression models indicated that there was significant heterogeneity in RSV percent positivity across subgroups defined by mid-year of the study period, WHO region, number of study sites, recruitment method, hospital type, and specimen type (*p* < 0.05).

**Conclusions:**

RSV detection was high among children aged < 5 years hospitalized with ARI in LMICs across all WHO regions, especially among infants aged < 6 months, among whom RSV may account for almost up to one-half of all ARI hospital admissions. Recent WHO-recommended RSV immunization for all countries may protect young infants aged < 6 months against severe RSV disease.

**Supplementary Information:**

The online version contains supplementary material available at 10.1186/s12889-026-26743-4.

## Introduction

RespiratorySyncytial Virus (RSV) is among the most prevalent causes of acute respiratory illness (ARI) in the paediatric population globally and causes severe disease in children of all income levels. In 2019, there were an estimated 101,400 RSV-attributable deaths in children aged < 5 years, representing 2.0% of all global childhood deaths and 3.6% of all deaths in children aged 28 days to 6 months [1]. Furthermore, RSV is associated with substantial morbidity and increased mortality in children with underlying conditions, such as prematurity, congenital heart disease, chronic lung disease, immunosuppression, and malnutrition. Overall, 46% of all RSV-attributable deaths occurred in children 0–5 months of age and 97% were in low- and middle-income countries (LMICs) [1]. Additionally, an estimated 3.6 million RSV-lower respiratory tract infection (LRTI) hospitalizations and 33 million RSV-LRTI episodes in young children occurred in 2019 [1]. In a large study of severe and very severe pneumonia in seven LMICs in Africa and Asia, RSV was the leading aetiology in all countries, accounting for 31% of cases overall [2].

After many decades of research and development, two highly effective immunization strategies against RSV have recently been authorized for use. RSV-PreF (Abrysvo©) is a protein subunit vaccine given to pregnant persons, leading to high-levels of transplacental transfer of anti-RSV antibodies, providing protection against severe RSV-LRTI through the first 6 months of life [3]. The second strategy is long-acting monoclonal antibodies, (nirsevimab (Beyfortus©; clesrovimab Eflonsia©), which are given to infants shortly before the RSV season begins, or within one week of birth for infants born during the RSV season, to provide passive immunization against severe RSV-LRTI for 5–6 months post-administration [4].

Although these new RSV products are currently available in many high-income countries (HIC), their use in LMICs has been very limited, due in part to their high cost. Gavi, the Vaccine Alliance cited limited knowledge of RSV disease burden in LMICs as a barrier to vaccine implementation [5]. Clinical testing for RSV is uncommon in most hospitals in LMICs, contributing to its under-recognition as a major cause of hospitalization [6]. Policymakers will therefore need to look to studies of national and/or regional disease burden data when considering the introduction of these new preventive products. To strengthen the global evidence base on severe RSV disease, we conducted a systematic review and meta-analysis of RSV-positivity among children aged < 5 years who were hospitalized for ARI in LMICs.

## Methods

### Registration and protocol

This systematic review and meta-analysis are reported according to PRISMA guidelines (Appendix 1, pp 2–6) [7]. The search strategy, inclusion criteria, and analysis are documented in a protocol registered with PROSPERO (registration ID: CRD42022361351).

### Search strategy and selection criteria

PubMed, Embase, Web of Science, and Scopus databases were searched using strategies with MeSH and Emtree terms without language restrictions for studies published between January 1, 2010 and October 14, 2022. Databases were searched for publications that included children aged < 5 years hospitalized for ARI and tested for RSV via polymerase chain reaction (PCR). For study eligibility purposes, we defined ARI as illnesses characterized by a sudden onset of respiratory symptoms such as cough, sore throat, shortness of breath, or coryza, with or without fever, in accordance with the World Health Organization (WHO) definition [8]. The search was supplemented by a bibliographic screening of papers yielded from our search that met study entry criteria. The search strategies and full search terms are available in Appendix 2 (pp 7–13) and in PROSPERO.

Study eligibility criteria are summarized in Appendix 3 (pp 14). Briefly, we included articles that reported acute hospitalization for ARI among children aged < 5 years in LMICs, used PCR for viral detection, and collected data in 2010 or after, but prior to the COVID-19 pandemic, when nonpharmaceutical interventions profoundly disrupted RSV transmission patterns [9]. Studies conducted exclusively among RSV-positive only patients, or that collected data only after 2019 were excluded, as were studies for which any inclusion criteria could not be disaggregated (e.g., results for children < 5 years were not disaggregated from that of older children). We also excluded studies with a surveillance period of < 12 calendar months or those with non-integer years of monitoring, considering the seasonality of RSV.

Titles and abstracts were screened for relevant publications by two teams of two graduate students. Discrepancies were discussed by the two respective screeners and, when a consensus could not be reached, a third, independent screener (CSL) resolved the conflict. If there was uncertainty over publication titles and abstracts, the publication(s) were included in the list for full-text screening. Full texts were screened for relevance by the same teams.

### Data extraction and quality assessment

Data were extracted using a standardized form in Microsoft Excel (version 2016; Microsoft Corporation, Redmond, USA). Disagreements were resolved through discussions with a third reviewer (HZ or MDK) and eligibility and key endpoints were reviewed and confirmed for all included studies (HZ or MDK). We systematically extracted the following data: first author’s name; publication date; study design; data collection date(s); country(ies); number of hospitalized children tested; number of positive test results; diagnostic methods; disease severity; and child age/group. Data from multicounty studies, or those that included multiple age groups, years of data collection, RSV seasons, or disease severity were extracted and presented by each respective group, where possible. Income level (low, lower-middle, upper-middle) was determined according to the World Bank classification at the time of data collection [10]. Two coauthors (HZ and HC) independently assessed the quality of the included studies using the Joanna Briggs Institute (JBI) Prevalence Critical Appraisal Tool, which is appropriate for proportion-based outcomes, with a third researcher (MDK) resolving any disagreements [11].

### Data analysis

We conducted a meta-analysis to estimate overall proportion positive for RSV and corresponding 95% confidence intervals (CIs) among children aged < 5 years hospitalized for ARI. We used the DerSimonian and Laird random-effects model (REM), which assumes that observed differences in RSV positivity reflect both real variation across studies and random error, and accounts for this by modelling both between- and within-study variance [12, 13]. We applied the Freeman-Tukey double arcsine transformation to the individual study proportions to stabilize the variance [14].

We reported overall LMIC estimates, as well as estimates stratified by income-level (low-, low-middle, upper-middle-income), WHO region (African Region [AFR], Region of the Americas [AMR], Eastern Mediterranean Region [EMR], European Region [EUR], South-East Asia Region [SEAR], Western Pacific Region [WPR]) [15], Human Development Index (HDI; low, medium, high, very high) [16], and age group (< 2 months, < 6 months, 6–11 months, < 12 months, and 6– < 60 months; some studies did not further characterize age beyond “ < 5 years” and were categorized as “ < 60 months, unspecified”).

We assessed between-study heterogeneity using the Q statistic and I^2^ test, considering I^2^> 75% indicative of high heterogeneity. We performed sensitivity analyses using the "leave-one-out" method to evaluate the influence of individual studies with large sample size on the global overall positivity estimate [17]. To investigate small-study effects, we applied Egger's test and visually examined the funnel plots, using the logit transformation of effect size and sample size [18]. To explore potential sources of heterogeneity, we conducted subgroup analyses and univariable meta-regression using random-effects models with restricted maximum likelihood estimation. A priori factors considered were mid-year of the study period, multiple/single year studied, WHO region, income level, HDI, study design, number of study sites, recruitment method, hospital type, specimen type, illness definition, and risk of bias.

We conducted all statistical analyses using Stata v17.0 (StataCorp, College Station, Texas, USA) and R version 4.3.1 (Bell Laboratories, Madison, WI, USA). Meta-analyses were conducted with the Stata “metaprop” procedure. We defined statistical significance as a *p*-value < 0.05.

### Role of the funding source

This work was supported by WHO through a grant from the Gates Foundation. Gates Foundation had no role in the development of this analysis.

## Results

**Fig. 1:**
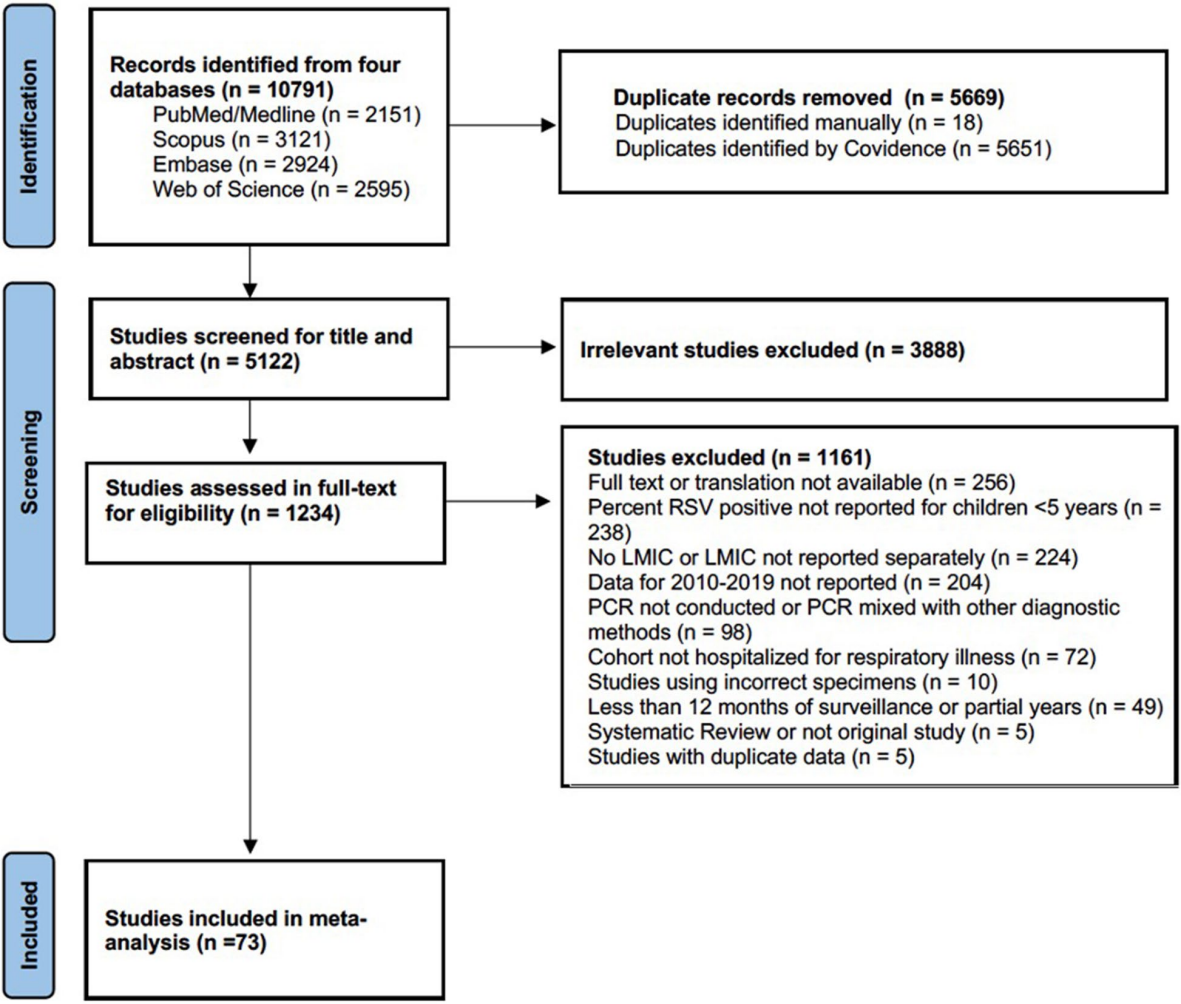
Literature search strategy and the numbers of included and excluded studies

Of 10,791 publications identified from database searches, 1,234 studies underwent full-text review, of which 73 studies were included, comprising data from 108,449 children < 60 months hospitalized with ARI who were tested for RSV (Fig. 1; Table 1; Appendix 4 pp 15–22). No additional publications were identified from the reference lists of included publications. Data were available from 37 countries in all WHO regions: 24 from WPR, 19 from AFR, 11 from SEAR, 11 from EMR, 4 from EUR, and 4 from AMR (Table 1). The country with the largest number of eligible studies was China (*n* = 16) (Table 1). Studies conducted in upper-middle-income countries (*n* = 45) were the most common. Most studies evaluated all children age < 5 years (*n* = 55); 11 included only children < 2 years, four < 1 year, one < 6 months, and two < 2 months of age. Additional study details are provided in Tables 1 and 2 and Appendix 4. Most (*n* = 51, 69.9%) studies were rated as low risk of bias, one as moderate risk (1.3%), and 21 (28.8%) as high risk (Appendix 4, pp 15–22). The most common reason for risk of bias was the inclusion of < 80% of eligible children aged < 60 months hospitalized with ARI, which if not selected randomly, could suggest insufficient representativeness of the target population. Assessment of funnel plots and results from Egger’s test (*p* = 0.62) indicated no evidence of publication bias (Appendix 5, pp 23–24).

**Table 1:** Global and regional percentage of RSV positivity among ARI hospital admissions in children aged < 5 years, by country

Country	Number of studies^a^	Number of children tested	Number of RSV positive children	Percentage positive^b^ % (95% CI)	Heterogeneity	
					**I**^**2**^	**Q**
Global		108449	31723	26.2 (24.3, 28.3)	98.1	9607.4
AFR	19	40308	10368	23.6 (21.3, 26.0)	96.8	1423.2
Cameroon	1	307	43	13.9 (10.2, 18.1)	-	-
Central African Republic	1	1231	164	13.3 (11.5, 15.3)	-	-
Côte d'Ivoire	1	1059	75	7.1 (5.6, 8.8)	-	-
Gambia	1	609	197	32.3 (28.6, 36.2)	-	-
Kenya	1	1433	363	25.3 (23.1, 27.6)	-	-
Madagascar	1	879	393	44.4 (41.1, 47.7)	-	-
Mali	1	768	194	25.2 (22.2, 28.3)	-	-
Mozambique	1	424	113	26.7 (22.5, 31.1)	-	-
Niger	2	1436	354	25.3 (17.1, 34.4)	91.9	49.3
South Africa	10	31206	8261	24.5 (21.9, 27.1)	96.5	735.9
Zambia	1	956	211	22.1 (19.5, 24.8)	-	-
AMR	4	4886	2896	34.8 (20.7, 50.4)	97.8	414.3
Argentina	1	3947	2588	65.6 (64.1, 67.1)	-	-
Colombia	1	419	147	34.8 (30.3, 39.5)	-	-
Haiti	1	101	26	25.7 (17.6, 35.4)	-	-
Panama	1	68	22	31.7 (21.0, 43.5)	-	-
Paraguay	1	99	14	14.1 (8.0, 22.6)	-	-
Suriname	1	252	99	35.8 (18.3, 55.5)	-	-
EMR	11	21788	8260	30.0 (25.1, 35.0)	98.1	1529.4
Egypt	2	4579	868	18.8 (17.3, 20.4)	44.0	5.4
Iran	1	158	49	29.3 (19.2, 40.6)	-	-
Iraq	1	468	106	17.2 (9.6, 26.2)	-	-
Jordan	4	15832	6983	43.1 (39.0, 47.3)	96.3	246.7
Lebanon	1	108	28	25.9 (18.0, 35.2)	-	-
Morocco	1	488	122	17.9 (8.3, 30.0)	-	-
Pakistan	1	155	104	67.1 (59.1, 74.4)	-	-
EUR	**4**	405	125	37.5 (19.1, 57.8)	91.2	57.1
Turkey	4	405	125	37.5 (19.1, 57.8)	91.2	57.1
SEAR	11	7484	1794	23.6 (19.7, 27.7)	93.4	409.3
Bangladesh	2	1251	275	21.4 (10.9, 34.2)	94.2	52.2
Bhutan	1	115	52	45.2 (35.9, 54.8)	-	-
India	6	4945	1189	22.3 (17.5, 27.6)	93.7	303.4
Thailand	2	1173	278	28.2 (18.1, 39.5)	92.2	25.8
WPR	24	33578	8280	26.0 (22.9, 29.2)	97.6	2358.6
Brazil	1	507	204	40.2 (35.9, 44.7)	-	-
Cambodia	1	176	39	22.2 (16.3,29.0)	-	-
China	16	22232	5342	24.5 (19.9, 29.4)	98.4	1847.7
Lao People's Democratic Republic	2	959	439	46.2 (38.5, 53.9)	80.5	20.5
Malaysia	1	284	99	34.8 (29.4, 40.5)	-	-
Mongolia	1	108	23	21.3 (14.0, 30.2)	-	-
Philippines	2	3393	801	24.1 (18.8, 29.8)	92.5	93.1
Vietnam	1	5919	1333	21.1 (17.5, 25.0)	-	-

*AFR* African Region, *AMR *Regions of the Americas, *ARI *acute respiratory infection, *CI *confidence interval, *EMR *Eastern Mediterranean Region, *EUR *European Region, *RSV *respiratory syncytial virus, *SEAR *South-East Asian Region, *WPR *Western Pacific Region, *Ref *reference group, *WHO *World Health Organization

^a^The global number of studies is not equal to the sum of studies across individual countries and regions, as some studies include data from multiple countries

^b^When only one study was conducted in a country, the percentage positive is that as reported by study authors; when two or more studies were conducted in a country, the percentage positive is the meta-analyzed estimate; the global estimate is the summary estimate of percent RSV-positive from the meta-analysis for all included studies.

**Table 2:** Overall percentage RSV-positive and association with study characteristics among ARI hospital admissions in children aged < 5 years

Variable subgroup	RSV positivity by subgroup			Meta-regression analysis		
	**No. of studies**	**No. children tested**	**Percentage positive % (95% CI)**	**OR**	**95% CI**	***P-*value**
Mid-year of the study period						
2010–2014	50	84,855	28.0 (25.6, 30.4)	Ref	Ref	**0.005**
2015–2019	23	23,594	22.3 (18.9, 25.8)	0.940	(0.899, 0.981)	
Multiple/Single year studied						
Multiple year studied	62	102,298	26.7 (24.6, 28.8)	Ref	Ref	0.16
Single year studied	11	6151	22.2 (17.6, 27.2)	0.953	(0.890, 1.017)	
WHO region						
AFR	19	40,308	23.6 (21.3, 26.0)	Ref	Ref	**0.007**
AMR	4	4886	34.8 (20.7, 50.4)	1.144	(1.032, 1.257)	
EMR	11	21,788	30.0 (25.1, 35.0)	1.092	(1.026, 1.157)	
EUR	4	405	37.5 (19.1, 57.8)	1.131	(0.957, 1.306)	
SEAR	11	7484	23.6 (19.7, 27.7)	1.003	(0.940, 1.065)	
WPR	24	33,578	26.0 (22.9, 29.2)	1.024	(0.975, 1.074)	
Income level						
Upper-middle income	45	77,059	28.3 (25.4, 31.2)	Ref	Ref	0.07
Low income	8	7258	25.5 (20.5, 30.8)	0.967	(0.899, 1.035)	
Lower-middle income	20	24,132	23.5 (21.0, 26.0)	0.951	(0.911, 0.992)	
Human Development Index (HDI)						
High	39	84,560	26.0 (23.7, 28.3)	Ref	Ref	0.13
Low	8	6561	26.7 (18.8, 35.5)	1.005	(0.930, 1.079)	
Medium	17	11,451	24.3 (21.2, 27.6)	0.990	(0.943, 1.038)	
Very high	9	5877	37.0 (23.1, 52.0)	1.108	(1.010, 1.206)	
Study design						
Sentinel surveillance	25	40,731	24.5 (21.7, 27.4)	Ref	Ref	0.60
Case–control	2	5479	25.6 (23.2, 28.1)	1.002	(0.912, 1.092)	
Cross-sectional	7	7426	27.9 (19.0, 37.6)	1.048	(0.956, 1.139)	
Prospective cohort	20	35,235	26.1 (22.5, 29.8)	1.023	(0.972, 1.074)	
Retrospective cohort	7	6776	26.2 (16.3, 37.5)	1.013	(0.92, 1.105)	
Not reported	12	12,802	29.4 (24.4, 34.6)	1.057	(0.991, 1.124)	
Number of study sites						
Multiple	31	58,712	24.3 (21.5, 27.2)	Ref	Ref	**0.04**
Single	41	49,494	28.1 (25.3, 31.0)	1.045	(1.003, 1.087)	
Not reported	1	243	13.1 (9.1, 17.7)	0.886	(0.704, 1.069)	
Recruitment method						
Sentinel surveillance	23	38,557	24.8 (21.7, 28.1)	Ref	Ref	**0.003**
Convenience sample	15	7317	27.4 (22.6, 32.4)	1.025	(0.964, 1.086)	
Random sample	2	1223	24.4 (6.4, 48.9)	0.976	(0.819, 1.134)	
Other^a^	5	11,255	40.3 (35.0, 45.7)	1.163	(1.072, 1.255)	
Not reported	28	50,097	24.5 (21.5, 27.7)	1.001	(0.955, 1.046)	
Hospital type						
Public only	33	48,989	29.6 (26.4, 33.0)	Ref	Ref	**0.02**
Private only	1	108	25.9 (18.0, 35.2)	0.952	(0.673, 1.232)	
Public and private	2	2202	20.0 (14.0, 26.7)	0.952	(0.673, 1.232)	
Not reported	37	57,150	23.6 (21.5, 25.7)	0.940	(0.902, 0.977)	
Specimen type						
Naso-oropharyngeal swab	18	31,927	28.9 (25.0, 33.0)	Ref	Ref	**0.002**
Nasopharyngeal swab	43	64,256	25.7 (23.1, 28.4)	0.961	(0.917, 1.005)	
Oropharyngeal swab	2	1293	9.7 (8.1, 11.4)	0.816	(0.713, 0.919)	
Other nasopharyngeal^b^	8	9023	31.7 (27.2, 36.4)	1.025	(0.952, 1.098)	
Other specimens^c^	2	1950	14.5 (0.073, 0.237)	0.874	(0.771, 0.977)	
Illness definition						
All ARI hospitalizations	50	71,357	26.9 (24.6, 29.2)	Ref	Ref	0.44
Severe ARI hospitalizations	23	37,092	25.0 (21.1, 29.0)	0.983	(0.941, 1.026)	
Risk of bias						
Low	51	82,389	25.0 (22.5, 27.7)	Ref	Ref	0.35
Moderate	1	52	37.5 (13.8, 64.4)	1.153	(0.803, 1.073)	
High	21	26,008	28.9 (26.6, 31.3)	1.027	(0.981, 1.073)	

*AFR* African Region, *AMR *Regions of the Americas, *ARI *acute respiratory infection, *CI *confidence interval, *EMR *Eastern Mediterranean Region, *EUR *European Region, *No *number, *SEAR *South-East Asian Region, *WPR *Western Pacific Region, *Ref *reference group, *WHO *World Health Organization

Boldface indicates statistical significance (*P*-value < 0.05)

^a^Other recruitment methods included enrolling the first two eligible children each day and implementing active surveillance

^b^Other nasopharyngeal included nasopharyngeal washings (NPWs), nasopharyngeal secretion specimens (NPSs), nasopharyngeal aspirates (NPAs), oropharyngeal and nasopharyngeal suctioning, nasopharyngeal aspirates combined with throat swabs, less invasive nasal (NS) samples

^c^Other specimens included induced sputum (IS), and tracheal secretions

RSV-positivity ranged from 7.1% (5.6–8.8%) in a Côte d'Ivoire study of children < 5 years to 67.1% (59.1–74.4%) in a Pakistan study of children < 2 years of age (inter-decile range from 11.0% to 48.1%; Appendix 4), with average percent RSV-positivity from meta-analysis of 26.2% (95% CI: 24.3–28.3%) and high between-study heterogeneity (I^2^ = 98.1%, Q = 9607.4, *p* < 0.001; Table 1). Sensitivity analyses that removed each of the five largest studies (32.2% of total children) produced similar results (range 25.9–26.6%; Appendix 6, pp 25).

**Fig. 2:**
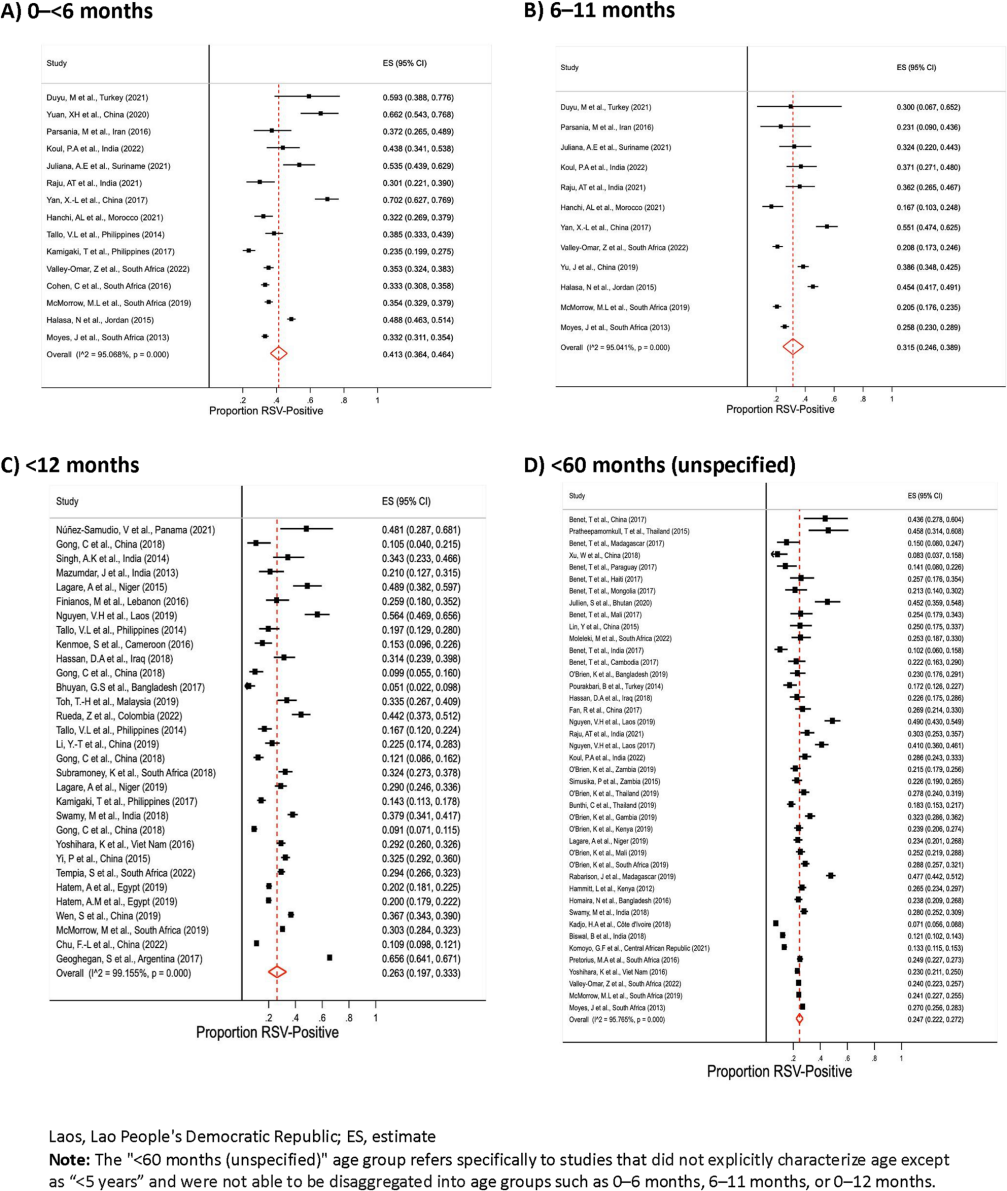
Global overall percentage of RSV-positivity among ARI hospital admissions in children < 5 years for select age groups. Laos, Lao People's Democratic Republic; ES, estimate. Note: The "<60 months (unspecified)" age group refers specifically to studies that did not explicitly characterize age except as “<5 years” and were not able to be disaggregated into age groups such as 0–6 months, 6–11 months, or 0–12 months. Panels A–D represent the following age groups: 0–<6 months (**A**), 6–11 months (**B**), <12 months (**C**), and <60 months (unspecified) (**D**)

**Fig. 3:**
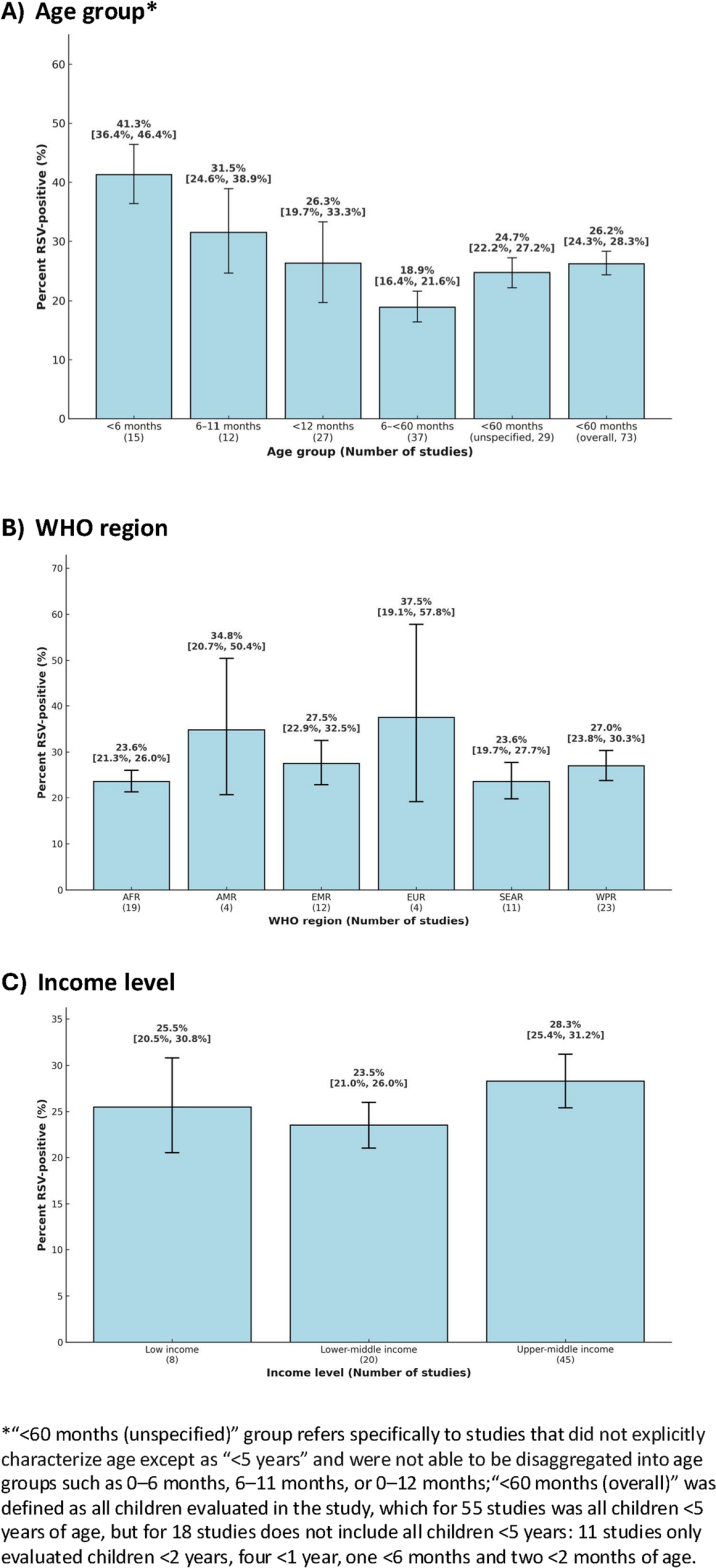
Global overall percentage of RSV-positivity among ARI hospital admissions in children < 5 years, stratified by WHO region, age group, and country income level. *“<60 months (unspecified)” group refers specifically to studies that did not explicitly characterize age except as “<5 years” and were not able to be disaggregated into age groups such as 0–6 months, 6–11 months, or 0–12 months;“<60 months (overall)” was defined as all children evaluated in the study, which for 55 studies was all children <5 years of age, but for 18 studies does not include all children <5 years: 11 studies only evaluated children <2 years, four <1 year, one <6 months and two <2 months of age. Panels A–C represent the following categories: age group (**A**), WHO region (**B**), and income level (**C**)

Children < 6 months of age had the highest RSV-positivity, which ranged from 23.5% to 70.2% across the 15 studies with data (meta-average: 41.3% [36.4—46.4%]; Figs. 2A and 3A); five studies (*n* = 40–840) that estimated positivity among children aged < 2 months had similar positivity (40.2% [35.8–44.7%]). RSV-positivity among children aged 6– < 60 months ranged from 1.4% to 66.0% (meta-average: 18.9% [16.4–21.6%]; Appendix 7, pp 26). Average percent RSV-positivity stratified by WHO region was highest in EUR (37.5% [19.1–57.8%]) and lowest in SEAR (23.6% [19.7–27.7%]) and AFR (23.6% [21.3–26.0%]; *p* = 0.007; Fig. 3B, Table 2). Additional meta-analysis results stratified by both WHO region and age group in the same analysis are presented in Appendix 8 (pp 27). There were minimal differences in the percentage RSV-positive by country income level: 28.3% (25.4–31.2%) in upper-middle-income countries (*n* = 45), 23.5% (21.0–26.0%) in lower-middle-income countries (*n* = 20), and 25.5% (20.5–30.8%) low-income countries (*n* = 8; *p* = 0.07; Fig. 3C). Other sources of heterogeneity where RSV-positivity differed included the year the study was conducted, number of study sites, recruitment method, type of hospital, and specimen type (Table 2*,* all *p* < 0.05).

## Discussion

In this systematic review and meta-analysis, we found that RSV was detected in over one-quarter of all children < 60 months hospitalized with ARI in LMIC settings. Across all six WHO regions, RSV was a major cause of hospitalization, ranging from 23.6% in SEARO and AFR to 37.5% in EURO, with minimal differences between countries of different income levels. RSV was most prevalent as a cause for ARI hospitalization among the youngest infants (41.3% among age < 6 months), the age group for whom the currently available prevention products – maternal RSV vaccine and infant long-acting monoclonal antibody – are targeted. Considering the high disease burden in the youngest infants, ensuring equitable access to these interventions is critical, especially in LMICs where RSV mortality is highest [1]. Results from this analysis may contribute to increasing awareness of RSV as a common cause of hospitalization among healthcare providers and vaccine policy makers with limited knowledge of local and/or regional RSV burden. Our results also highlight the importance of preventing RSV among older children 6– < 60 months of age, who also experience a substantial burden of hospitalization due to RSV, but for whom prevention products are not currently available. Future vaccines aimed at preventing RSV disease in toddlers are in late-stage development [19, 20].

Our results are consistent with other recent estimates of the burden of RSV among children hospitalized with ARI. In the PERCH study, which enrolled over 4,000 severe and very severe pneumonia cases in seven countries in Africa and Asia, RSV was the leading etiology, identified in 31.3% of all cases in children < 5 years; among infants aged < 12 months, RSV was identified as the etiology in 39.7% of cases [2]. Additionally, in the Drakenstein South Africa birth cohort study, over 1,000 infants were followed from birth to 2 years of age [21]. Hospitalization for LRTI was common, occurring at an incidence of 0.08 per child-year; RSV accounted for one-third of all hospitalizations [22]. In a separate multi-country birth cohort study, approximately 2% of Bangladeshi children and 1% of Thai and Argentinean children were hospitalized with RSV-LRTI before their second birthday [23]. Most recently, the RSV Gold-ICU multi-country network found that among over 2,000 children < 24 months admitted to a paediatric ICU with LRTI, 29.0% were RSV positive, the majority of whom were aged < 6 months [24]. We also found substantial heterogeneity across the included studies, reflecting not only biological and epidemiological differences (e.g., age distribution and seasonality) but also considerable variation in hospital admission thresholds, healthcare access, RSV testing practices, and diagnostic capacity across countries and regions.

The largest previous systematic review and meta-analysis that evaluated global RSV morbidity and mortality burden reported RSV-positivity among hospitalized children as a secondary result [1]. This analysis included 122 studies from LMICs and reported RSV positivity was 23–26% among children < 60 months, approximately 25% for children < 12 months, and 32–33% for children < 6 months. Our estimates in children < 60 months and < 12 months (both 26.3%) are consistent; however, our estimate for children < 6 months is higher (41.3%). This difference may be due to methodological differences between our analysis and the previous study. First, the analysis conducted by Li et al. included studies published from 2015–2019 only, whereas the current study included those published between 2010–2022. Additionally, the search strategy from Li and colleagues required RSV as a primary diagnosis, whereas our review required hospitalization with ARI in which patients were tested for RSV and did not stipulate a specific diagnosis. Li and colleagues allowed for diagnostic methods other than PCR (whereas we excluded any non-PCR laboratory methods) and excluded studies with fewer than 50 patients (whereas we did not exclude studies based on patient number). Finally, 27 (22%) of the studies included by Li et al. were conducted in HICs, and they did not stratify results by WHO region.

This review only included studies from the pre-COVID-19 era to quantify and describe RSV disease burden under typical epidemiological conditions before viral transmission patterns were interrupted during the pandemic. The impact of COVID-19 mitigation measures (e.g., masking, social distancing) has been shown to have significantly decreased RSV transmission, leading to extremely low incidence of RSV globally [25–27]. Furthermore, after the relaxation of COVID-19 policies, RSV resurgence occurred outside established seasonality, in higher rates than pre-COVID-19, and resulted in more severe disease among older children who were not exposed to RSV in infancy [26, 27]. These atypical patterns of RSV epidemiology during the pandemic would misrepresent RSV burden and were therefore excluded from this review. As of 2024, RSV epidemiology has largely returned to pre-pandemic patterns, and continuing to estimate burden will be critical to future prevention efforts.

This study was subject to limitations. First, not all WHO regions were represented equally and there was a comparatively low number of studies from EUR and AMR (4 studies each, compared to 11–24 in other regions). The limited number of studies in the AMR and EUR region is likely due to having few LMICs in these two regions, among which few surveillance studies met our inclusion criteria. Additionally, some regional data was represented by a low number of individual countries. For example, all studies among children < 6 months in AFR were conducted in South Africa, whose sociodemographic and economic profile is different from other countries in the region. Second, detection of RSV is not always indicative of causation, and co-infections or nosocomial acquisition of RSV might overestimate assessment of the hospitalized RSV burden. However, previous studies have reported that the attributable fraction of RSV among RSV-positive hospitalizations is high (≥ 90%) [28, 29]. Finally, the percentage positive among ARI hospitalizations likely does not represent the full burden of severe RSV disease in these settings: healthcare utilization and testing practices may be limited in LMICs, thereby a sizeable proportion of children with severe RSV disease may not make it to a hospital or be identified as RSV-positive if admitted [6].

Estimates from this analysis can guide decision-making regarding target populations and optimal timing of administration [30]. The high RSV-positivity in children aged < 2 months indicate the importance of protection from birth, either through maternal immunization or administration of RSV monoclonal antibodies in the first few days of life. Despite its documented burden in all regions globally as shown in our work, RSV is often under-recognized as a cause of severe ARI in LMICs. Generating more country and regional evidence regarding RSV burden in hospital and community settings, its seasonality, and high-risk groups is critical to inform immunization policies and facilitate programmatic implementation. Demonstrating a high disease burden strengthens the case for investing in immunization programs. In 2017, 14 countries piloted the integration of RSV surveillance into the Global Influenza Surveillance Response System (GISRS) in anticipation of data needs as prevention products came to market [31, 32]; as of 2025, this strategy has been implemented in 25 total countries [33].

## Conclusions

This systematic review and meta-analysis highlight the substantial burden of RSV-associated ARI hospitalizations across all WHO regions, with particularly high burden among infants < 6 months of age. These findings reinforce the global public health imperative to make RSV immunization accessible to LMICs, where RSV burden on fragile health system capacity can be substantial. While country-specific data remain valuable for decision-making, the evidence generated through this review provide a strong rationale for LMICs to adopt RSV immunization policies now. Delaying implementation until national surveillance for RSV is fully established could result in missed opportunities to prevent severe RSV-associated disease and death.

## Supplementary Information

Supplementary Material 1.

## Data Availability

All data analysed are available within the publications listed in Appendix 4 (pp 15-22).

## Abbreviations

RSV: Respiratory syncytial virus
LMIC: Low- and middle-income countries
CI: Confidence interval
ARI: Acute respiratory illness
LRTI: Lower respiratory tract infection
HIC: High-income countries
PCR: Polymerase chain reaction
WHO: World Health Organization
REM: Random-effects model
AFR: African Region
AMR: Region of the Americas
EMR: Eastern Mediterranean Region
EUR: European Region
SEAR: South-East Asia Region
WPR: Western Pacific Region
GISRS: Global Influenza Surveillance Response System
