# Supplementary material for: Respiratory syncytial virus positivity among hospital admissions for acute respiratory illness in children younger than 5 years of age in low- and middle-income countries: a systematic review and meta-analysis
Source: BMC Public Health. 2026 Feb 24;26:1059. doi: 10.1186/s12889-026-26743-4 (PMC13037220; doi:10.1186/s12889-026-26743-4)
Supplement: Supplementary file 1 — Supplementary Material 1. [file 12889_2026_26743_MOESM1_ESM.docx]

**Supplementary appendixes**

**Respiratory syncytial virus positivity among hospital admissions for acute respiratory illness in children younger than 5 years of age in low- and middle-income countries: a systematic review and meta-analysis**

Contents

[Appendix 1. PRISMA (Preferred Reporting Items for Systematic Reviews and Meta-Analyses) checklist of this review 2](#_Toc206250443)

[Appendix 2. Search strategy used in each database 7](#_Toc206250444)

[Appendix 3. Eligibility criteria for the systematic review and meta-analysis 14](#_Toc206250445)

[Appendix 4. Key characteristics of eligible studies included in our study 15](#_Toc206250446)

[Appendix 5. Funnel plots used to assess publication bias 23](#_Toc206250447)

[Appendix 6. Leave-one-out meta-analysis for large sample size study sensitivity analysis 25](#_Toc206250448)

[Appendix 7. Global overall percentage of RSV-positivity among ARI hospital admissions in children aged 6 months-5 years 26](#_Toc206250449)

[Appendix 8. Supplemental meta-analysis results stratified by WHO regions and age group 27](#_Toc206250450)

[Appendix 9. Global distribution of under five children RSV positive proportion eligible, peer-reviewed studies worldwide—based on data published from January 1, 2010 to October 14, 2022 28](#_Toc206250451)

# Appendix 1. PRISMA (Preferred Reporting Items for Systematic Reviews and Meta-Analyses) checklist of this review

| **Section and Topic** | **Item #** | **Checklist item** | **Location where item is reported** |
| --- | --- | --- | --- |
| **TITLE** | | |  |
| Title | 1 | Identify the report as a systematic review. | 1 |
| **ABSTRACT** | | |  |
| Abstract | 2 | See the PRISMA 2020 for Abstracts checklist. | 2 |
| **INTRODUCTION** | | |  |
| Rationale | 3 | Describe the rationale for the review in the context of existing knowledge. | 3 |
| Objectives | 4 | Provide an explicit statement of the objective(s) or question(s) the review addresses. | 5 |
| **METHODS** | | |  |
| Eligibility criteria | 5 | Specify the inclusion and exclusion criteria for the review and how studies were grouped for the syntheses. | 6; Appendix 3 |
| Information sources | 6 | Specify all databases, registers, websites, organisations, reference lists and other sources searched or consulted to identify studies. Specify the date when each source was last searched or consulted. | 6 |
| Search strategy | 7 | Present the full search strategies for all databases, registers and websites, including any filters and limits used. | Appendix 2 |
| Selection process | 8 | Specify the methods used to decide whether a study met the inclusion criteria of the review, including how many reviewers screened each record and each report retrieved, whether they worked independently, and if applicable, details of automation tools used in the process. | 6-7 |
| Data collection process | 9 | Specify the methods used to collect data from reports, including how many reviewers collected data from each report, whether they worked independently, any processes for obtaining or confirming data from study investigators, and if applicable, details of automation tools used in the process. | 6 |
| Data items | 10a | List and define all outcomes for which data were sought. Specify whether all results that were compatible with each outcome domain in each study were sought (e.g. for all measures, time points, analyses), and if not, the methods used to decide which results to collect. | 6-7 |
|  | 10b | List and define all other variables for which data were sought (e.g. participant and intervention characteristics, funding sources). Describe any assumptions made about any missing or unclear information. | 6-7 |
| Study risk of bias assessment | 11 | Specify the methods used to assess risk of bias in the included studies, including details of the tool(s) used, how many reviewers assessed each study and whether they worked independently, and if applicable, details of automation tools used in the process. | 7 |
| Effect measures | 12 | Specify for each outcome the effect measure(s) (e.g. risk ratio, mean difference) used in the synthesis or presentation of results. | 7 |
| Synthesis methods | 13a | Describe the processes used to decide which studies were eligible for each synthesis (e.g. tabulating the study intervention characteristics and comparing against the planned groups for each synthesis (item #5)). | 6-7 |
|  | 13b | Describe any methods required to prepare the data for presentation or synthesis, such as handling of missing summary statistics, or data conversions. | 6-7 |
|  | 13c | Describe any methods used to tabulate or visually display results of individual studies and syntheses. | 6-7 |
|  | 13d | Describe any methods used to synthesize results and provide a rationale for the choice(s). If meta-analysis was performed, describe the model(s), method(s) to identify the presence and extent of statistical heterogeneity, and software package(s) used. | 6-7 |
|  | 13e | Describe any methods used to explore possible causes of heterogeneity among study results (e.g. subgroup analysis, meta-regression). | 7 |
|  | 13f | Describe any sensitivity analyses conducted to assess robustness of the synthesized results. | 6-7 |
| Reporting bias assessment | 14 | Describe any methods used to assess risk of bias due to missing results in a synthesis (arising from reporting biases). | 6-7 |
| Certainty assessment | 15 | Describe any methods used to assess certainty (or confidence) in the body of evidence for an outcome. | 6-7 |
| **RESULTS** | | |  |
| Study selection | 16a | Describe the results of the search and selection process, from the number of records identified in the search to the number of studies included in the review, ideally using a flow diagram. | Figure 1 |
|  | 16b | Cite studies that might appear to meet the inclusion criteria, but which were excluded, and explain why they were excluded. | Figure 1 |
| Study characteristics | 17 | Cite each included study and present its characteristics. | Appendix 4 |
| Risk of bias in studies | 18 | Present assessments of risk of bias for each included study. | Appendix 4 |
| Results of individual studies | 19 | For all outcomes, present, for each study: (a) summary statistics for each group (where appropriate) and (b) an effect estimate and its precision (e.g. confidence/credible interval), ideally using structured tables or plots. | 8-9 |
| Results of syntheses | 20a | For each synthesis, briefly summarise the characteristics and risk of bias among contributing studies. | 9 |
|  | 20b | Present results of all statistical syntheses conducted. If meta-analysis was done, present for each the summary estimate and its precision (e.g. confidence/credible interval) and measures of statistical heterogeneity. If comparing groups, describe the direction of the effect. | 8-9 |
|  | 20c | Present results of all investigations of possible causes of heterogeneity among study results. | 9 |
|  | 20d | Present results of all sensitivity analyses conducted to assess the robustness of the synthesized results. | 9 |
| Reporting biases | 21 | Present assessments of risk of bias due to missing results (arising from reporting biases) for each synthesis assessed. | 8 |
| Certainty of evidence | 22 | Present assessments of certainty (or confidence) in the body of evidence for each outcome assessed. | 9 |
| **DISCUSSION** | | |  |
| Discussion | 23a | Provide a general interpretation of the results in the context of other evidence. | 9-10 |
|  | 23b | Discuss any limitations of the evidence included in the review. | 11 |
|  | 23c | Discuss any limitations of the review processes used. | 11 |
|  | 23d | Discuss implications of the results for practice, policy, and future research. | 11-12 |
| **OTHER INFORMATION** | | |  |
| Registration and protocol | 24a | Provide registration information for the review, including register name and registration number, or state that the review was not registered. | 5 |
|  | 24b | Indicate where the review protocol can be accessed, or state that a protocol was not prepared. | 5 |
|  | 24c | Describe and explain any amendments to information provided at registration or in the protocol. | 5 |
| Support | 25 | Describe sources of financial or non-financial support for the review, and the role of the funders or sponsors in the review. | 8 |
| Competing interests | 26 | Declare any competing interests of review authors. | 12 |
| Availability of data, code and other materials | 27 | Report which of the following are publicly available and where they can be found: template data collection forms; data extracted from included studies; data used for all analyses; analytic code; any other materials used in the review. | 12 |

*From:*  Page MJ, McKenzie JE, Bossuyt PM, Boutron I, Hoffmann TC, Mulrow CD, et al. The PRISMA 2020 statement: an updated guideline for reporting systematic reviews. BMJ 2021;372:n71. doi: 10.1136/bmj.n71. This work is licensed under CC BY 4.0. To view a copy of this license, visit <https://creativecommons.org/licenses/by/4.0/> ^1^

# Appendix 2. Search strategy used in each database

| **PUBMED** | | **Strategy steps** |
| --- | --- | --- |
| 1 | ((("Respiratory Syncytial Virus, Human"[Mesh]) OR ("Human Respiratory Syncytial Virus"[Title/Abstract])) OR ("Respiratory Syncytial Virus"[Title/Abstract])) OR ("RSV"[Title/Abstract]) | #1 |
| 2 | (("Child, Preschool"[Mesh]) OR ("Preschool Children"[Title/Abstract])) OR ("Preschool Child"[Title/Abstract]) | #2 |
| 3 | (("Infant"[Mesh]) OR ("Infants"[Title/Abstract])) OR ("Infant"[Title/Abstract]) | #3 |
| 4 | ((("Infant, Newborn"[Mesh]) OR ("Newborn Infant*"[Title/Abstract])) OR ("Newborn*"[Title/Abstract])) OR ("Neonate*"[Title/Abstract]) | #4 |
| 5 | (("Hospitalization"[Mesh]) OR ("Hospitalization*"[Title/Abstract])) OR ("Hospitalized"[Title/Abstract]) | #5 |
| 6 | ((("Respiratory Syncytial Virus Infections"[Mesh]) OR ("Respiratory Syncytial Virus Infection*"[Title/Abstract])) OR ("RSV Infection*"[Title/Abstract])) OR (RSV[Title/Abstract]) | #6 |
| 7 | ("Child, Hospitalized"[Mesh]) OR ("Hospitalized Child*"[Title/Abstract]) | #7 |
| 8 | ((((("Intensive Care Units, Pediatric"[Mesh]) OR (Pediatric Intensive Care Unit*[Title/Abstract])) OR (Paediatric Intensive Care Unit*[Title/Abstract])) OR (Pediatric ICU*[Title/Abstract])) OR (ICU, Pediatric[Title/Abstract])) OR (ICUs, Pediatric[Title/Abstract]) | #8 |
| 9 | (("Respiratory Tract Infections"[Mesh]) OR (Respiratory Tract Infection*[Title/Abstract]) OR (Respiratory Infection*[Title/Abstract])) OR ((Respiratory Tract Infection*[Title/Abstract]) OR (Respiratory Infection*[Title/Abstract])) | #9 |
| 10 | ((((("Virus Diseases"[Mesh]) OR (Virus Disease*[Title/Abstract])) OR (Virus Infection*[Title/Abstract])) OR (Viral Disease*[Title/Abstract])) OR (Viral Infection*[Title/Abstract])) AND ((RSV[Title/Abstract]) OR (respiratory syncytial virus[Title/Abstract])) | #10 |
| 11 | ((("Respiratory Tract Infections"[Mesh]) OR (Respiratory Tract Infection*[Title/Abstract]) OR (Respiratory Infection*[Title/Abstract])) OR ((Respiratory Tract Infection*[Title/Abstract]) OR (Respiratory Infection*[Title/Abstract]))) AND (((((("Virus Diseases"[Mesh]) OR (Virus Disease*[Title/Abstract])) OR (Virus Infection*[Title/Abstract])) OR (Viral Disease*[Title/Abstract])) OR (Viral Infection*[Title/Abstract])) AND ((RSV[Title/Abstract]) OR (respiratory syncytial virus[Title/Abstract]))) | #9 AND #10 |
| 12 | (((("Respiratory Syncytial Virus Infections"[Mesh]) OR ("Respiratory Syncytial Virus Infection*"[Title/Abstract])) OR ("RSV Infection*"[Title/Abstract])) OR (RSV[Title/Abstract])) OR (((("Respiratory Tract Infections"[Mesh]) OR (Respiratory Tract Infection*[Title/Abstract]) OR (Respiratory Infection*[Title/Abstract])) OR ((Respiratory Tract Infection*[Title/Abstract]) OR (Respiratory Infection*[Title/Abstract]))) AND (((((("Virus Diseases"[Mesh]) OR (Virus Disease*[Title/Abstract])) OR (Virus Infection*[Title/Abstract])) OR (Viral Disease*[Title/Abstract])) OR (Viral Infection*[Title/Abstract])) AND (RSV[Title/Abstract]) OR ((respiratory syncytial virus[Title/Abstract]))) | #6 OR #11 |
| 13 | (((("Child, Preschool"[Mesh]) OR ("Preschool Children"[Title/Abstract])) OR ("Preschool Child"[Title/Abstract])) OR ((("Infant"[Mesh]) OR ("Infants"[Title/Abstract])) OR ("Infant"[Title/Abstract]))) OR (((("Infant, Newborn"[Mesh]) OR ("Newborn Infant*"[Title/Abstract])) OR ("Newborn*"[Title/Abstract])) OR ("Neonate*"[Title/Abstract])) | #2 OR #3 OR #4 |
| 14 | ((((("Child, Preschool"[Mesh]) OR ("Preschool Children"[Title/Abstract])) OR ("Preschool Child"[Title/Abstract])) OR ((("Infant"[Mesh]) OR ("Infants"[Title/Abstract])) OR ("Infant"[Title/Abstract]))) OR (((("Infant, Newborn"[Mesh]) OR ("Newborn Infant*"[Title/Abstract])) OR ("Newborn*"[Title/Abstract])) OR ("Neonate*"[Title/Abstract]))) AND ((("Hospitalization"[Mesh]) OR ("Hospitalization*"[Title/Abstract])) OR ("Hospitalized"[Title/Abstract])) | #13 AND #15 |
| 15 | ((("Child, Hospitalized"[Mesh]) OR ("Hospitalized Child*"[Title/Abstract])) OR (((((("Intensive Care Units, Pediatric"[Mesh]) OR (Pediatric Intensive Care Unit*[Title/Abstract])) OR (Paediatric Intensive Care Unit*[Title/Abstract])) OR (Pediatric ICU*[Title/Abstract])) OR (ICU, Pediatric[Title/Abstract])) OR (ICUs, Pediatric[Title/Abstract]))) OR (((((("Child, Preschool"[Mesh]) OR ("Preschool Children"[Title/Abstract])) OR ("Preschool Child"[Title/Abstract])) OR ((("Infant"[Mesh]) OR ("Infants"[Title/Abstract])) OR ("Infant"[Title/Abstract]))) OR (((("Infant, Newborn"[Mesh]) OR ("Newborn Infant*"[Title/Abstract])) OR ("Newborn*"[Title/Abstract])) OR ("Neonate*"[Title/Abstract]))) AND ((("Hospitalization"[Mesh]) OR ("Hospitalization*"[Title/Abstract])) OR ("Hospitalized"[Title/Abstract]))) | #7 OR #8 OR #14 |
| 16 | ((((("Respiratory Syncytial Virus Infections"[Mesh]) OR ("Respiratory Syncytial Virus Infection*"[Title/Abstract])) OR ("RSV Infection*"[Title/Abstract])) OR (RSV[Title/Abstract])) OR (((("Respiratory Tract Infections"[Mesh]) OR (Respiratory Tract Infection*[Title/Abstract]) OR (Respiratory Infection*[Title/Abstract])) OR ((Respiratory Tract Infection*[Title/Abstract]) OR (Respiratory Infection*[Title/Abstract]))) AND (((((("Virus Diseases"[Mesh]) OR (Virus Disease*[Title/Abstract])) OR (Virus Infection*[Title/Abstract])) OR (Viral Disease*[Title/Abstract])) OR (Viral Infection*[Title/Abstract])) AND ((RSV[Title/Abstract]) OR (respiratory syncytial virus[Title/Abstract))))) AND (((("Child, Hospitalized"[Mesh]) OR ("Hospitalized Child*"[Title/Abstract])) OR (((((("Intensive Care Units, Pediatric"[Mesh]) OR (Pediatric Intensive Care Unit*[Title/Abstract])) OR (Paediatric Intensive Care Unit*[Title/Abstract])) OR (Pediatric ICU*[Title/Abstract])) OR (ICU, Pediatric[Title/Abstract])) OR (ICUs, Pediatric[Title/Abstract]))) OR (((((("Child, Preschool"[Mesh]) OR ("Preschool Children"[Title/Abstract])) OR ("Preschool Child"[Title/Abstract])) OR ((("Infant"[Mesh]) OR ("Infants"[Title/Abstract])) OR ("Infant"[Title/Abstract]))) OR (((("Infant, Newborn"[Mesh]) OR ("Newborn Infant*"[Title/Abstract])) OR ("Newborn*"[Title/Abstract])) OR ("Neonate*"[Title/Abstract]))) AND ((("Hospitalization"[Mesh]) OR ("Hospitalization*"[Title/Abstract])) OR ("Hospitalized"[Title/Abstract])))) | #12 AND #15 |
| 17 | ((((("Respiratory Syncytial Virus Infections"[Mesh]) OR ("Respiratory Syncytial Virus Infection*"[Title/Abstract])) OR ("RSV Infection*"[Title/Abstract])) OR (RSV[Title/Abstract])) OR (((("Respiratory Tract Infections"[Mesh]) OR (Respiratory Tract Infection*[Title/Abstract]) OR (Respiratory Infection*[Title/Abstract])) OR ((Respiratory Tract Infection*[Title/Abstract]) OR (Respiratory Infection*[Title/Abstract]))) AND (((((("Virus Diseases"[Mesh]) OR (Virus Disease*[Title/Abstract])) OR (Virus Infection*[Title/Abstract])) OR (Viral Disease*[Title/Abstract])) OR (Viral Infection*[Title/Abstract])) AND ((RSV[Title/Abstract]) OR (respiratory syncytial virus[Title/Abstract))))) AND (((("Child, Hospitalized"[Mesh]) OR ("Hospitalized Child*"[Title/Abstract])) OR (((((("Intensive Care Units, Pediatric"[Mesh]) OR (Pediatric Intensive Care Unit*[Title/Abstract])) OR (Paediatric Intensive Care Unit*[Title/Abstract])) OR (Pediatric ICU*[Title/Abstract])) OR (ICU, Pediatric[Title/Abstract])) OR (ICUs, Pediatric[Title/Abstract]))) OR (((((("Child, Preschool"[Mesh]) OR ("Preschool Children"[Title/Abstract])) OR ("Preschool Child"[Title/Abstract])) OR ((("Infant"[Mesh]) OR ("Infants"[Title/Abstract])) OR ("Infant"[Title/Abstract]))) OR (((("Infant, Newborn"[Mesh]) OR ("Newborn Infant*"[Title/Abstract])) OR ("Newborn*"[Title/Abstract])) OR ("Neonate*"[Title/Abstract]))) AND ((("Hospitalization"[Mesh]) OR ("Hospitalization*"[Title/Abstract])) OR ("Hospitalized"[Title/Abstract])))) AND 2010[dp] : "now"[dp] | #16 AND  RESTRICT TO 2010 AND MORE RECENT |

| **EMBASE** | | **Strategy steps** |
| --- | --- | --- |
| 1 | child'/exp OR 'child':ab,kw,ti | #1 |
| 2 | infant'/exp OR 'infant*':ab,kw,ti | #2 |
| 3 | newborn'/exp OR 'newborn*':ab,kw,ti | #3 |
| 4 | hospital patient'/exp OR 'hospitalized':ab,kw,ti OR 'intensive care'/exp OR 'ICU':ab,kw,ti | #4 |
| 5 | respiratory syncytial virus infection'/exp OR 'respiratory syncytial virus infection*':ab,kw,ti OR 'RSV infection':ab,kw,ti OR 'RSV':ab,kw,ti | #5 |
| 6 | hospitalized child'/exp OR 'hospitalized infant'/exp | #6 |
| 7 | newborn intensive care'/exp OR 'pediatric intensive care unit*':ab,kw,ti OR 'paediatric intensive care unit*':ab,kw,ti OR 'PICU':ab,kw,ti | #7 |
| 8 | ('respiratory tract infection'/exp OR 'viral respiratory tract infection'/exp OR 'respiratory tract infection*':ab,kw,ti OR 'respiratory infection*':ab,kw,ti) AND ('respiratory syncytial virus':ab,kw,ti OR 'RSV':ab,kw,ti) | #8 |
| 9 | ('child'/exp OR 'child':ab,kw,ti) OR ('infant'/exp OR 'infant*':ab,kw,ti) OR ('newborn'/exp OR 'newborn*':ab,kw,ti) | #1 OR #2 OR #3 |
| 10 | ('hospitalized child'/exp OR 'hospitalized infant'/exp ) OR ('newborn intensive care'/exp OR 'pediatric intensive care unit*':ab,kw,ti OR 'paediatric intensive care unit*':ab,kw,ti OR 'PICU':ab,kw,ti) | #10 |
| 11 | (('child'/exp OR 'child':ab,kw,ti) OR ('infant'/exp OR 'infant*':ab,kw,ti) OR ('newborn'/exp OR 'newborn*':ab,kw,ti)) AND ('hospital patient'/exp OR 'hospitalized':ab,kw,ti OR 'intensive care'/exp OR 'ICU':ab,kw,ti) | #4 AND #9 |
| 12 | ('respiratory syncytial virus infection'/exp OR 'respiratory syncytial virus infection*':ab,kw,ti OR 'RSV infection':ab,kw,ti OR 'RSV':ab,kw,ti) OR (('respiratory tract infection'/exp OR 'viral respiratory tract infection'/exp OR 'respiratory tract infection*':ab,kw,ti OR 'respiratory infection*':ab,kw,ti) AND ('respiratory syncytial virus':ab,kw,ti OR 'RSV':ab,kw,ti)) | #5 OR #8 |
| 13 | ((('child'/exp OR 'child':ab,kw,ti) OR ('infant'/exp OR 'infant*':ab,kw,ti) OR ('newborn'/exp OR 'newborn*':ab,kw,ti)) AND ('hospital patient'/exp OR 'hospitalized':ab,kw,ti OR 'intensive care'/exp OR 'ICU':ab,kw,ti)) OR (('hospitalized child'/exp OR 'hospitalized infant'/exp ) OR ('newborn intensive care'/exp OR 'pediatric intensive care unit*':ab,kw,ti OR 'paediatric intensive care unit*':ab,kw,ti OR 'PICU':ab,kw,ti)) | #6 OR #11 |
| 14 | (('respiratory syncytial virus infection'/exp OR 'respiratory syncytial virus infection*':ab,kw,ti OR 'RSV infection':ab,kw,ti OR 'RSV':ab,kw,ti) OR (('respiratory tract infection'/exp OR 'viral respiratory tract infection'/exp OR 'respiratory tract infection*':ab,kw,ti OR 'respiratory infection*':ab,kw,ti) AND ('respiratory syncytial virus':ab,kw,ti OR 'RSV':ab,kw,ti))) AND (((('child'/exp OR 'child':ab,kw,ti) OR ('infant'/exp OR 'infant*':ab,kw,ti) OR ('newborn'/exp OR 'newborn*':ab,kw,ti)) AND ('hospital patient'/exp OR 'hospitalized':ab,kw,ti OR 'intensive care'/exp OR 'ICU':ab,kw,ti)) OR (('hospitalized child'/exp OR 'hospitalized infant'/exp ) OR ('newborn intensive care'/exp OR 'pediatric intensive care unit*':ab,kw,ti OR 'paediatric intensive care unit*':ab,kw,ti OR 'PICU':ab,kw,ti))) | #12 AND #13 |
| 15 | ('respiratory syncytial virus infection'/exp OR 'respiratory syncytial virus infection*':ab,kw,ti OR 'rsv infection':ab,kw,ti OR 'rsv':ab,kw,ti OR (('respiratory tract infection'/exp OR 'viral respiratory tract infection'/exp OR 'respiratory tract infection*':ab,kw,ti OR 'respiratory infection*':ab,kw,ti) AND ('respiratory syncytial virus':ab,kw,ti OR 'rsv':ab,kw,ti))) AND (('child'/exp OR 'child':ab,kw,ti OR 'infant'/exp OR 'infant*':ab,kw,ti OR 'newborn'/exp OR 'newborn*':ab,kw,ti) AND ('hospital patient'/exp OR 'hospitalized':ab,kw,ti OR 'intensive care'/exp OR 'icu':ab,kw,ti) OR 'hospitalized child'/exp OR 'hospitalized infant'/exp OR 'newborn intensive care'/exp OR 'pediatric intensive care unit*':ab,kw,ti OR 'paediatric intensive care unit*':ab,kw,ti OR 'picu':ab,kw,ti) AND [2010-2022]/py | #14 AND RESTRICT TO 2010 AND MORE RECENT |

| **SCOPUS** | | **Strategy steps** |
| --- | --- | --- |
| 1 | TITLE-ABS-KEY (child OR infant OR newborn OR neonate) | #1 |
| 2 | TITLE-ABS-KEY ( hospitalized OR hospitalization OR icu OR "intensive care unit*" ) | #2 |
| 3 | TITLE-ABS-KEY ( picu OR nicu OR "pediatric intensive care unit*" OR "paediatric intensive care unit*" OR "neonatal intensive care unit*" ) | #3 |
| 4 | TITLE-ABS-KEY ( ( child OR infant OR newborn OR neonate ) AND ( hospitalized OR hospitalization OR icu OR "intensive care unit*" ) ) | #1 AND #2 |
| 5 | TITLE-ABS-KEY ( ( ( child OR infant OR newborn OR neonate ) AND ( hospitalized OR hospitalization OR icu OR "intensive care unit*" ) ) OR ( picu OR nicu OR "pediatric intensive care unit*" OR "paediatric intensive care unit*" OR "neonatal intensive care unit*" ) ) | #3 AND #4 |
| 6 | TITLE-ABS-KEY ( rsv OR "respiratory syncytial virus infection*" OR "RSV infection*" ) | #6 |
| 7 | TITLE-ABS-KEY ( ( rti OR "respiratory tract infection*" OR "respiratory infection*" ) AND ( rsv OR "respiratory syncytial virus" ) ) | #7 |
| 8 | TITLE-ABS-KEY ( ( rsv OR "respiratory syncytial virus infection*" OR "RSV infection*" ) OR ( ( rti OR "respiratory tract infection*" OR "respiratory infection*" ) AND ( rsv OR "respiratory syncytial virus" ) ) ) | #6 OR #7 |
| 9 | TITLE-ABS-KEY ( ( ( rsv OR "respiratory syncytial virus infection*" OR "RSV infection*" ) OR ( ( rti OR "respiratory tract infection*" OR "respiratory infection*" ) AND ( rsv OR "respiratory syncytial virus" ) ) ) AND ( ( ( child OR infant OR newborn OR neonate ) AND ( hospitalized OR hospitalization OR icu OR "intensive care unit*" ) ) OR ( picu OR nicu OR "pediatric intensive care unit*" OR "paediatric intensive care unit*" OR "neonatal intensive care unit*" ) ) ) | #5 AND #8 |
| 10 | TITLE-ABS-KEY ( ( ( rsv OR "respiratory syncytial virus infection*" OR "RSV infection*" ) OR ( ( rti OR "respiratory tract infection*" OR "respiratory infection*" ) AND ( rsv OR "respiratory syncytial virus" ) ) ) AND ( ( ( child OR infant OR newborn OR neonate ) AND ( hospitalized OR hospitalization OR icu OR "intensive care unit*" ) ) OR ( picu OR nicu OR "pediatric intensive care unit*" OR "paediatric intensive care unit*" OR "neonatal intensive care unit*" ) ) ) AND ( LIMIT-TO ( PUBYEAR , 2023 ) OR LIMIT-TO ( PUBYEAR , 2022 ) OR LIMIT-TO ( PUBYEAR , 2021 ) OR LIMIT-TO ( PUBYEAR , 2020 ) OR LIMIT-TO ( PUBYEAR , 2019 ) OR LIMIT-TO ( PUBYEAR , 2018 ) OR LIMIT-TO ( PUBYEAR , 2017 ) OR LIMIT-TO ( PUBYEAR , 2016 ) OR LIMIT-TO ( PUBYEAR , 2015 ) OR LIMIT-TO ( PUBYEAR , 2014 ) OR LIMIT-TO ( PUBYEAR , 2013 ) OR LIMIT-TO ( PUBYEAR , 2012 ) OR LIMIT-TO ( PUBYEAR , 2011 ) OR LIMIT-TO ( PUBYEAR , 2010 ) ) | #9 AND RESTRICT TO 2010 AND MORE RECENT |

| **WEB OF SCIENCE** | | **Strategy steps** |
| --- | --- | --- |
| 1 | TS=(((rsv OR "respiratory syncytial virus infection*" OR "RSV infection*") OR ((rti OR "respiratory tract infection*" OR "respiratory infection*") AND (rsv OR "respiratory syncytial virus"))) AND (((child OR infant OR newborn OR neonate) AND (hospitalized OR hospitalization OR icu OR "intensive care unit*")) OR (picu OR nicu OR "pediatric intensive care unit*" OR "paediatric intensive care unit*" OR "neonatal intensive care unit*"))) | #1 |
| 2 | TS=(((rsv OR "respiratory syncytial virus infection*" OR "RSV infection*") OR ((rti OR "respiratory tract infection*" OR "respiratory infection*") AND (rsv OR "respiratory syncytial virus"))) AND (((child OR infant OR newborn OR neonate) AND (hospitalized OR hospitalization OR icu OR "intensive care unit*")) OR (picu OR nicu OR "pediatric intensive care unit*" OR "paediatric intensive care unit*" OR "neonatal intensive care unit*"))) AND PY=(2010-2022) | #1 AND RESTRICT TO 2010 AND MORE RECENT |

# Appendix 3. Eligibility criteria for the systematic review and meta-analysis

| **Inclusion:** |  |
| --- | --- |
|  | 1. Human children younger than 5 years hospitalized for an acute respiratory illness (any severity)  2. Children must be tested for Respiratory Syncytial Virus (RSV) via polymerase chain reaction (PCR)  3. At the time of data collection (per study), country/ies must be low- or middle-income status  4. At least 12 months of surveillance; no partial years |
| **Exclusion:** |  |
|  | 1. Studies that only include persons five years or older, or studies where data for children younger than five years cannot be disaggregated from older subjects 2. Only RSV-positive children enrolled or only virus positive children enrolled 3. PCR testing for RSV not conducted or PCR testing mixed with other diagnostic methods 4. Study conducted in high-income country/ies only 5. Data collection prior to 2010 only (i.e., no data collected prior to COVID-19 pandemic) or data collection after 2019 only |

# Appendix 4. Key characteristics of eligible studies included in our study

| **Author (Year)/References** | **Country** | **WHO region** | **Age group** | **Income level** | **Study design** | **HDI level** | **Specimen type** | **Recruitment method** | **Site type** | **Risk of bias** | **# Children tested** | **# Positive hospitalized children** | **Percentage positive, %** |
| --- | --- | --- | --- | --- | --- | --- | --- | --- | --- | --- | --- | --- | --- |
| Bashir, U et al.(2017)^2^ | Pakistan | EMR | <2 years | Lower-middle | Sentinel surveillance | Low | Naso-oro-pharyngeal swab | Sentinel surveillance | Multiple | High | 155 | 104 | 67.10 |
| Benet, T et al.(2017)^3^ | Cambodia | WPR | <5 years | Low | Prospective cohort | Medium | Nasopharyngeal swab | Convenience sample | Single | Low | 888 | 178 | 20.05 |
| Bhuyan, G.S et al.(2017)^4^ | Bangladesh | SEAR | <1 year; > 6 months | Lower-middle | Prospective cohort | Medium | Nasopharyngeal swab | Convenience sample | Multiple | Low | 200 | 27 | 13.50 |
| Biswal, B et al.(2018)^5^ | India | SEAR | <5 years | Lower-middle | Cross-sectional | Medium | Naso-oro-pharyngeal swab | Random sample | Multiple | Low | 1063 | 129 | 12.14 |
| Bunthi, C et al.(2019)^6^ | Thailand | SEAR | <5 years | Upper-middle | Sentinel surveillance | Very High | Tracheal secretion; lung tissues; Nasopharyngeal swab | Sentinel surveillance | Multiple | Low | 600 | 110 | 18.33 |
| Chu, F.-L et al.(2022)^7^ | China | WPR | <1 year; > 6 months | Upper-middle | Retrospective cohort | High | Nasopharyngeal swab | Not reported | Single | Low | 3873 | 384 | 9.91 |
| Cohen, C et al.(2016)^8^ | South Africa | AFR | <6 months | Upper-middle | Prospective cohort | High | Nasopharyngeal swab | Sentinel surveillance | Multiple | High | 1410 | 469 | 33.26 |
| Duyu, M et al.(2021)^9^ | Turkey | EUR | <6 months; 6-11 months; > 6 months | Upper-middle | Prospective cohort | Very High | Tracheal and nasopharyngeal swabs | Not reported | Single | Middle | 52 | 22 | 42.31 |
| Fadugba, O.O et al.(2021)^10^ | Jordan | EMR | <2 years | Upper-middle | Cross-sectional | High | Naso-oro-pharyngeal swab | Not reported | Single | Low | 3168 | 1397 | 44.10 |
| Fan, R et al.(2017)^11^ | China | WPR | <5 years | Upper-middle | Sentinel surveillance | High | Nasopharyngeal swab | Sentinel surveillance | Single | Low | 238 | 64 | 26.89 |
| Finianos, M et al.(2016)^12^ | Lebanon | EMR | <1 year | Upper-middle | Not reported | High | Nasopharyngeal swab | Convenience sample | Single | High | 108 | 28 | 25.93 |
| Geoghegan, S et al.(2017)^13^ | Argentina | AMR | <1 year | Upper-middle | Prospective cohort | Very High | Nasopharyngeal swab | Not reported | Multiple | Low | 3947 | 2588 | 65.57 |
| Gong, C et al.(2018)^14^ | China | WPR | <1 year | Upper-middle | Sentinel surveillance | High | Oropharyngeal swab | Sentinel surveillance | Multiple | Low | 1197 | 120 | 10.03 |
| Gurgel, R.Q et al.(2016)^15^ | Brazil | WPR | <2 years | Upper-middle | Cross-sectional | High | Nasopharyngeal swab | Convenience sample | Multiple | Low | 507 | 204 | 40.24 |
| Halasa, N et al.(2015)^16^ | Jordan | EMR | 0-2 months; <6 months; 6-11 months; > 6 months | Upper-middle | Sentinel surveillance | High | Naso-oro-pharyngeal swab | Sentinel surveillance | Single | Low | 3973 | 1610 | 40.52 |
| Hanchi, AL et al.(2021)^17^ | Morocco | EMR | <6 months; 6-11 months; > 6 months | Lower-middle | Retrospective cohort | Medium | Nasopharyngeal swab | Not reported | Single | Low | 488 | 122 | 25.00 |
| Hassan, D.A et al.(2018)^18^ | Iraq | EMR | <1 year; > 6 months; <5 years | Upper-middle | Prospective cohort | Medium | Nasopharyngeal swab | Not reported | Single | Low | 468 | 106 | 22.65 |
| Hatem, A et al.(2019)^19^ | Egypt | EMR | <1 year; > 6 months | Lower-middle | Not reported | High | Naso-oro-pharyngeal swab | Not reported | Single | Low | 2283 | 434 | 19.01 |
| Hatem, A.M et al.(2019)^20^ | Egypt | EMR | <1 year; > 6 months | Lower-middle | Sentinel surveillance | High | Naso-oro-pharyngeal swab | Sentinel surveillance | Single | Low | 2296 | 434 | 18.90 |
| Homaira, N et al.(2016)^21^ | Bangladesh | SEAR | <5 years | Low | Sentinel surveillance | Medium | Naso-oro-pharyngeal swab | Sentinel surveillance | Multiple | Low | 829 | 197 | 23.76 |
| Juliana, A.E et al.(2021)^22^ | Suriname | AMR | <6 months; 6-11 months; > 6 months | Upper-middle | Sentinel surveillance | Medium | Nasopharyngeal swab | Sentinel surveillance | Single | Low | 252 | 99 | 39.29 |
| Jullien, S et al.(2020)^23^ | Bhutan | SEAR | <5 years | Lower-middle | Prospective cohort | Medium | Nasopharyngeal washing (NPW) | Not reported | Single | High | 115 | 52 | 45.22 |
| Kadjo, H.A et al.(2018)^24^ | Côte d'Ivoire | AFR | <5 years | Lower-middle | Sentinel surveillance | Low | Nasopharyngeal swab | Sentinel surveillance | Multiple | Low | 1059 | 75 | 7.08 |
| Kamigaki, T et al.(2017)^25^ | Philippines | WPR | <6 months; <1 year; > 6 months | Lower-middle | Sentinel surveillance | High | Naso-oro-pharyngeal swab | Convenience sample | Multiple | High | 2053 | 409 | 19.92 |
| Karabay, M et al.(2022)^26^ | Turkey | EUR | 0-2 months | Upper-middle | Retrospective cohort | Very High | Nasopharyngeal swab | Not reported | Single | Low | 40 | 18 | 45.00 |
| Kenmoe, S et al.(2016)^27^ | Cameroon | AFR | <1 year; > 6 months | Lower-middle | Prospective cohort | Medium | Nasopharyngeal swab | Convenience sample | Single | Low | 307 | 43 | 14.01 |
| Khuri-Bulos, N et al.(2018)^28^ | Jordan | EMR | <2 years | Upper-middle | Prospective cohort | High | Naso-oro-pharyngeal swab | Sentinel surveillance | Single | Low | 3168 | 1397 | 44.10 |
| Klink, T et al.(2020)^29^ | Jordan | EMR | <2 years | Upper-middle | Prospective cohort | High | Naso-oro-pharyngeal swab | Active surveillance | Single | Low | 6328 | 2792 | 44.12 |
| Komoyo, G.F et al.(2021)^30^ | Central African Republic | AFR | <5 years | Low | Sentinel surveillance | Low | Nasopharyngeal swab | Sentinel surveillance | Multiple | Low | 1231 | 164 | 13.32 |
| Koul, P.A et al.(2022)^31^ | India | SEAR | <6 months; 6-11 months; > 6 months; <5 years | Lower-middle | Prospective cohort | Medium | Nasopharyngeal swab | Enroll the first 2 children eligible everyday | Multiple | High | 824 | 236 | 28.64 |
| Lagare, A et al.(2015)^32^ | Niger | AFR | <1 year; > 6 months | Low | Retrospective cohort | Low | Nasopharyngeal swab | Random sample | Multiple | High | 160 | 56 | 35.00 |
| Lagare, A et al.(2019)^33^ | Niger | AFR | <1 year; > 6 months; <5 years | Low | Prospective cohort | Low | Nasopharyngeal swab | Not reported | Multiple | Low | 1276 | 298 | 23.35 |
| Li, Y.-T et al.(2019)^34^ | China | WPR | <1 year; > 6 months | Upper-middle | Not reported | High | Nasopharyngeal aspirate (NPA) or induced sputum (IS) | Not reported | Single | Low | 545 | 73 | 13.39 |
| Lin, Y et al.(2015)^35^ | China | WPR | <5 years | Upper-middle | Not reported | High | Nasopharyngeal swab | Not reported | Single | Low | 120 | 30 | 25.00 |
| Liu, T et al.(2015)^36^ | China | WPR | <2 years; > 6 months | Upper-middle | Not reported | High | Naso-oro-pharyngeal swab | Convenience sample | Not Reported | Low | 243 | 32 | 13.17 |
| Luo, H.-J et al.(2020)^37^ | China | WPR | <2 years | Upper-middle | Not reported | High | Nasopharyngeal swab | Not reported | Multiple | Low | 5659 | 1273 | 22.50 |
| Mazumdar, J et al.(2013)^38^ | India | SEAR | <1 year; > 6 months | Lower-middle | Cross-sectional | Medium | Naso-oro-pharyngeal swab | Not reported | Multiple | Low | 108 | 18 | 16.67 |
| McMorrow, M et al.(2019)^39^ | South Africa | AFR | <1 year | Upper-middle | Sentinel surveillance | High | Nasopharyngeal swab | Not reported | Multiple | High | 2243 | 680 | 30.32 |
| McMorrow, M.L et al.(2019)^40^ | South Africa | AFR | 0-2 months; <6 months; 6-11 months; > 6 months; <5 years | Upper-middle | Sentinel surveillance | High | Nasopharyngeal swab | Sentinel surveillance | Multiple | Low | 8102 | 2074 | 25.60 |
| Moleleki, M et al.(2022)^41^ | South Africa | AFR | <5 years | Upper-middle | Sentinel surveillance | High | Nasopharyngeal swab | Sentinel surveillance | Multiple | High | 154 | 39 | 25.32 |
| Moyes, J et al.(2013)^42^ | South Africa | AFR | <6 months; 6-11 months; > 6 months; <5 years | Upper-middle | Sentinel surveillance | High | Nasopharyngeal swab | Sentinel surveillance | Multiple | High | 8585 | 2314 | 26.95 |
| Nguyen, V.H et al.(2017)^43^ | Lao People's Democratic Republic | WPR | <5 years | Lower-middle | Prospective cohort | Medium | Naso-oro-pharyngeal swab | Convenience sample | Single | Low | 383 | 157 | 40.99 |
| Nguyen, V.H et al.(2019)^44^ | Lao People's Democratic Republic | WPR | <1 year; > 6 months; <5 years | Lower-middle | Not reported | Medium | Nasopharyngeal swabs (NPS), less invasive nasal (NS) and throat (TS) swabs | Not reported | Single | Low | 576 | 282 | 48.96 |
| Núñez-Samudio, V et al.(2021)^45^ | Panama | AMR | <1 year; > 6 months | Upper-middle | Retrospective cohort | Very High | Nasopharyngeal swab | Not reported | Single | Low | 68 | 22 | 32.35 |
| O'Brien, K et al.(2019)^46^ | Gambia | AFR | <5 years | Low | Case-control | Low | Naso-oro-pharyngeal swab | Not reported | Multiple | High | 3889 | 1036 | 26.64 |
| Okulu, E et al.(2018)^47^ | Turkey | EUR | 0-2 months | Upper-middle | Retrospective cohort | Very High | Nasopharyngeal swab | Convenience sample | Single | High | 81 | 45 | 55.56 |
| Pale, M et al.(2017)^48^ | Mozambique | AFR | <2 years | Low | Sentinel surveillance | Low | Nasopharyngeal swab | Sentinel surveillance | Single | High | 424 | 113 | 26.65 |
| Parsania, M et al.(2016)^49^ | Iran (Islamic Republic of) | EMR | <6 months; 6-11 months; > 6 months | Upper-middle | Not reported | High | Naso-oro-pharyngeal swab | Convenience sample | Single | Low | 158 | 49 | 31.01 |
| Pourakbari, B et al.(2014)^50^ | Turkey | EUR | <5 years | Upper-middle | Sentinel surveillance | Very High | Nasopharyngeal swab | Sentinel surveillance | Single | Low | 232 | 40 | 17.24 |
| Pratheepamornkull, T et al.(2015)^51^ | Thailand | SEAR | <5 years | Upper-middle | Cross-sectional | Very High | Nasopharyngeal swab | Convenience sample | Single | High | 48 | 22 | 45.83 |
| Pretorius, M.A et al.(2016)^52^ | South Africa | AFR | <5 years | Upper-middle | Sentinel surveillance | High | Nasopharyngeal swab | Sentinel surveillance | Multiple | Low | 1431 | 357 | 24.95 |
| Rabarison, J et al.(2019)^53^ | Madagascar | AFR | <5 years | Low | Sentinel surveillance | Low | Naso-oro-pharyngeal swab | All patients meeting the SARI case definition were eligible for enrollment. | Single | High | 799 | 381 | 47.68 |
| Raju, AT et al.(2021)^54^ | India | SEAR | <6 months; 6-11 months; > 6 months; <5 years | Lower-middle | Prospective cohort | Medium | Nasal and throat swabs | Not reported | Single | High | 634 | 192 | 30.28 |
| Ren, K.-Y et al.(2021)^55^ | China | WPR | <2 years | Upper-middle | Retrospective cohort | High | Oropharyngeal and Nasopharyngeal Suctioning | Not reported | Single | Low | 2066 | 826 | 39.98 |
| Rueda, Z et al.(2022)^56^ | Colombia | AMR | <1 year; > 6 months | Upper-middle | Prospective cohort | High | Nasopharyngeal swab | Not reported | Multiple | High | 419 | 147 | 35.08 |
| Simusika, P et al.(2015)^57^ | Zambia | AFR | <5 years | Lower-middle | Sentinel surveillance | Medium | Naso-oro-pharyngeal swab | Sentinel surveillance | Single | Low | 496 | 112 | 22.58 |
| Singh, A.K et al.(2014)^58^ | India | SEAR | <1 year; > 6 months | Lower-middle | Prospective cohort | Medium | Nasopharyngeal swab | Convenience sample | Single | Low | 155 | 39 | 25.16 |
| Subramoney, K et al.(2018)^59^ | South Africa | AFR | <1 year; > 6 months | Upper-middle | Prospective cohort | High | Nasopharyngeal swab | Sentinel surveillance | Multiple | Low | 462 | 125 | 27.06 |
| Swamy, M et al.(2018)^60^ | India | SEAR | <1 year; > 6 months; <5 years | Lower-middle | Not reported | Medium | Nasopharyngeal aspirate and throat swabs | Not reported | Single | Low | 1994 | 558 | 27.98 |
| Tallo, V.L et al.(2014)^61^ | Philippines | WPR | <1 year; > 6 months | Lower-middle | Sentinel surveillance | High | Naso-oro-pharyngeal swab | Sentinel surveillance | Multiple | High | 1340 | 392 | 29.25 |
| Tempia, S et al.(2022)^62^ | South Africa | AFR | <1 year; > 6 months | Upper-middle | Sentinel surveillance | High | Nasopharyngeal swab | Sentinel surveillance | Multiple | Low | 1632 | 379 | 23.22 |
| Toh, T.-H et al.(2019)^63^ | Malaysia | WPR | <1 year; > 6 months | Upper-middle | Not reported | Very High | Nasopharyngeal swab | Convenience sample | Multiple | Low | 284 | 99 | 34.86 |
| Valley-Omar, Z et al.(2022)^64^ | South Africa | AFR | 0-2 months; <6 months; 6-11 months; > 6 months; <5 years | Upper-middle | Prospective cohort | High | Nasopharyngeal swab | Not reported | Multiple | Low | 5607 | 1431 | 25.52 |
| Wen, S et al.(2019)^65^ | China | WPR | <1 year; > 6 months | Upper-middle | Prospective cohort | High | Nasopharyngeal secretion specimens (NPSs) | Not reported | Single | Low | 2673 | 871 | 32.59 |
| Xu, L et al.(2012)^66^ | China | WPR | <2 years | Upper-middle | Sentinel surveillance | High | Throat swabs | Not reported | Multiple | Low | 913 | 203 | 22.23 |
| Xu, W et al.(2018)^67^ | China | WPR | <5 years | Upper-middle | Sentinel surveillance | High | Oropharyngeal swab | Sentinel surveillance | Single | High | 96 | 8 | 8.33 |
| Yan, X.-L et al.(2017)^68^ | China | WPR | <6 months; 6-11 months; > 6 months | Upper-middle | Not reported | High | Nasopharyngeal swab | Convenience sample | Single | Low | 696 | 359 | 51.58 |
| Yi, P et al.(2015)^69^ | China | WPR | <1 year; > 6 months | Upper-middle | Cross-sectional | High | Nasopharyngeal swab | Not reported | Single | Low | 1267 | 331 | 26.12 |
| Yoshihara, K et al.(2016)^70^ | Viet Nam | WPR | <1 year; > 6 months; <5 years | Lower-middle | Prospective cohort | High | Nasopharyngeal swab | Not reported | Single | Low | 5919 | 1333 | 22.52 |
| Yu, J et al.(2018)^71^ | China | WPR | <2 years | Upper-middle | Sentinel surveillance | High | Nasopharyngeal swab | Convenience sample | Multiple | High | 1206 | 303 | 25.12 |
| Yu, J et al.(2019)^72^ | China | WPR | 6-11 months; > 6 months | Upper-middle | Cross-sectional | High | Nasopharyngeal swab | Not reported | Single | High | 1265 | 369 | 29.17 |
| Yuan, XH et al.(2020)^73^ | China | WPR | <6 months; > 6 months | Upper-middle | Not reported | High | Nasopharyngeal swab | Patients were enrolled within 24 hours of admission | Single | Low | 136 | 79 | 58.09 |
| Zar, H.J et al.(2020)^74^ | South Africa | AFR | <2 years | Upper-middle | Case-control | High | Nasopharyngeal swab | Not reported | Single | Low | 785 | 164 | 20.89 |

**Note:** AFR, African Region; AMR, Regions of the Americas; ARI, acute respiratory infection; CI, confidence interval; EMR, Eastern Mediterranean Region; EUR, European Region; No., number; SEAR, South East Asian Region; WPR, West Pacific Region.

# Appendix 5. Funnel plots used to assess publication bias


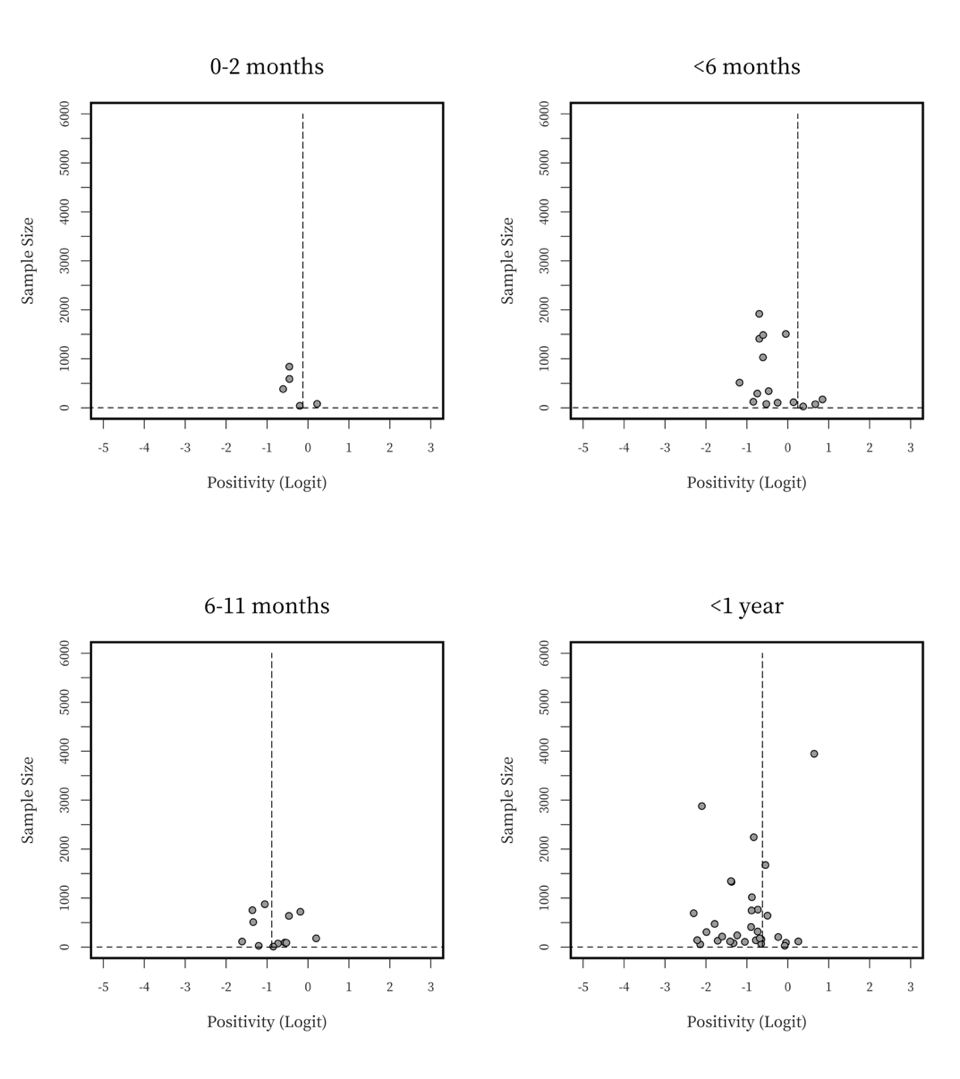


**Note:** The vertical dashed line represents the overall positivity estimate from the meta-analysis.


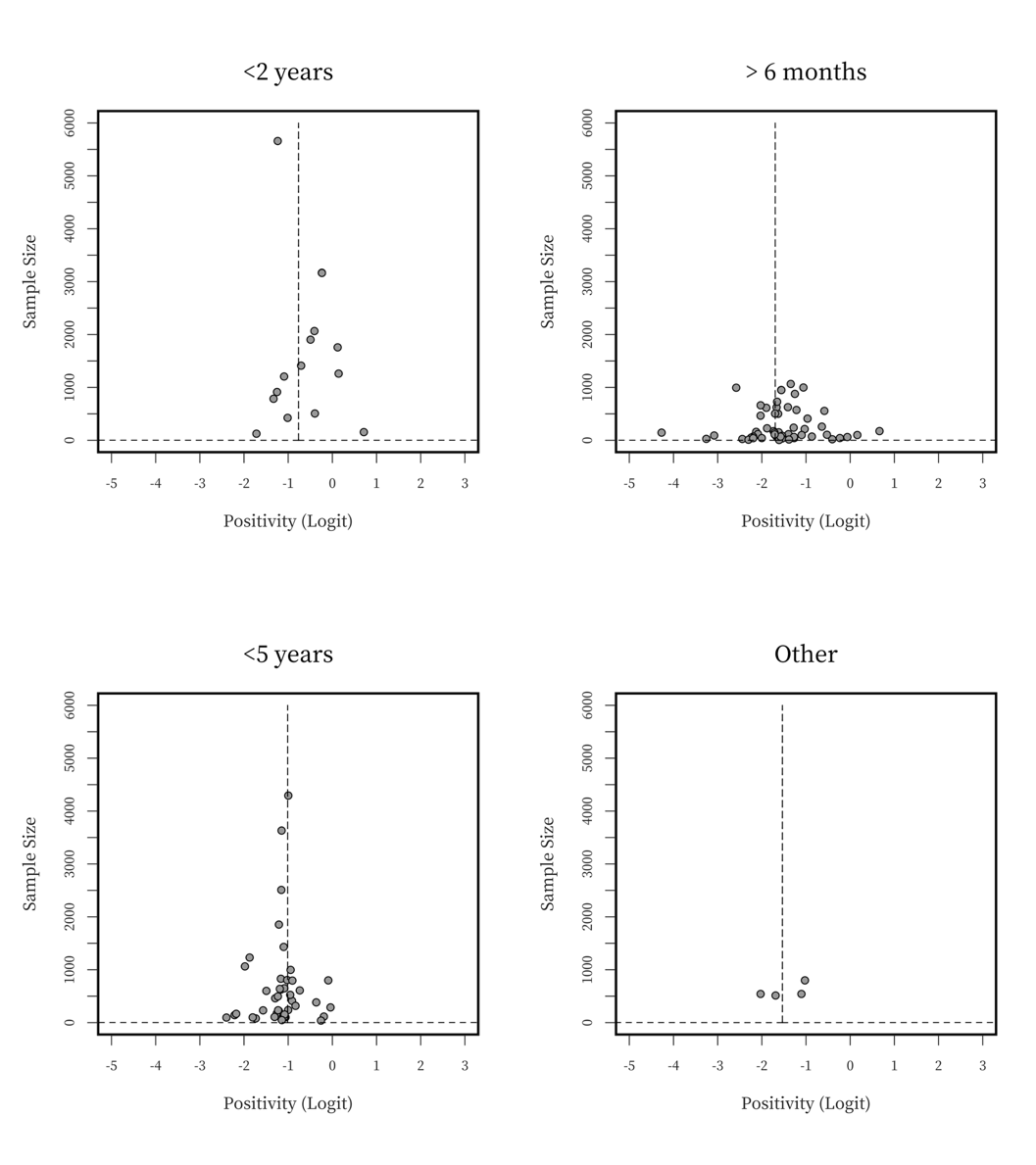


These funnel plots indicated no such bias was detected for the studies included in the present review and meta-analysis.

**Note:** The vertical dashed line represents the overall positivity estimate from the meta-analysis.

# Appendix 6. Leave-one-out meta-analysis for large sample size study sensitivity analysis

| **Author (Year)/References** | **Country** | **Number of children**  **tested (total)** | **Number of RSV**  **positive children** | **Percentage positive %**  **(95% CI)** | **Heterogeneity** | |
| --- | --- | --- | --- | --- | --- | --- |
|  |  |  |  |  | **I^2^** | **Q** |
| **Omitted five studies with**  **the largest sample size** | |  |  |  |  |  |
| Moyes, J et al.(2013)^42^ | South Africa | 8585 | 2314 | 26.3 (24.2, 28.4) | 98.18 | 9502.5 |
| McMorrow, M.L et al.(2019)^40^ | South Africa | 8102 | 2074 | 26.3 (24.3, 28.5) | 98.15 | 9281.3 |
| Klink, T et al.(2020)^29^ | Jordan | 6328 | 2792 | 25.9 (23.9, 27.9) | 98.02 | 8813.9 |
| Yoshihara, K et al.(2016)^70^ | Vietnam | 5919 | 1333 | 26.6 (24.5, 28.7) | 98.20 | 9391.3 |
| Luo, H.-J et al.(2020)^37^ | China | 5659 | 1273 | 26.3 (24.2, 28.4) | 98.14 | 9495.1 |

# Appendix 7. Global overall percentage of RSV-positivity among ARI hospital admissions in children aged 6 months-5 years


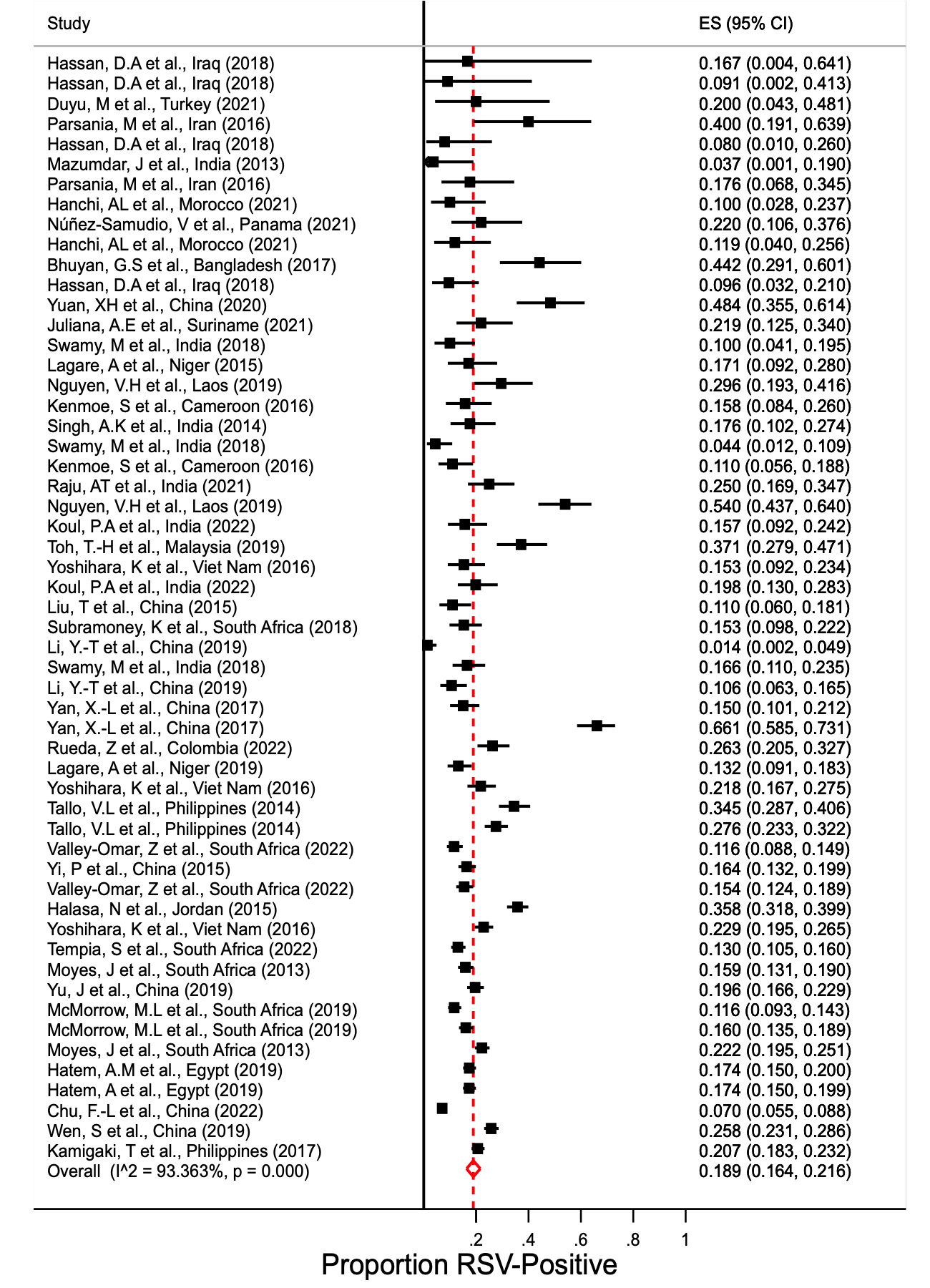


**Note:** Laos, Lao People's Democratic Republic; ES, estimate

# Appendix 8. Supplemental meta-analysis results stratified by WHO regions and age group

1. **Meta analysis results stratified by WHO regions (< 6 months)**


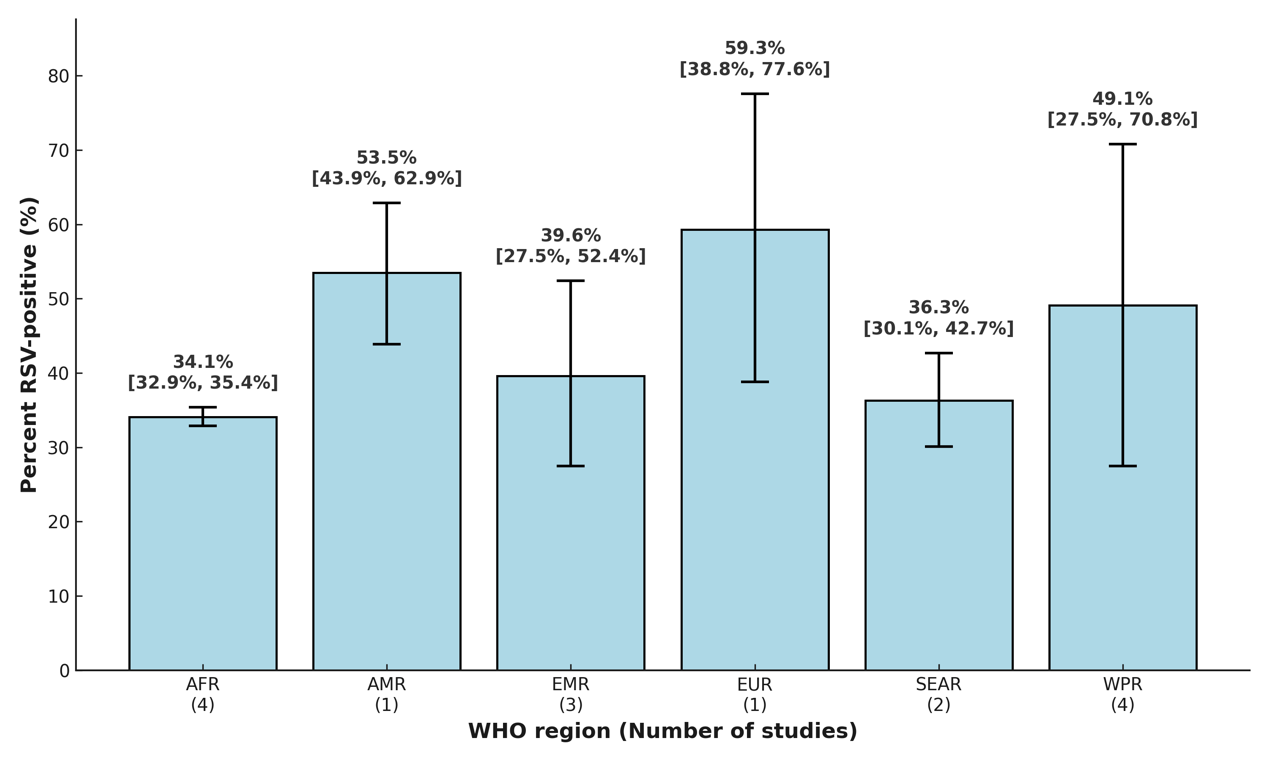


1. **Meta analysis results stratified by WHO regions (0–<60 months)**


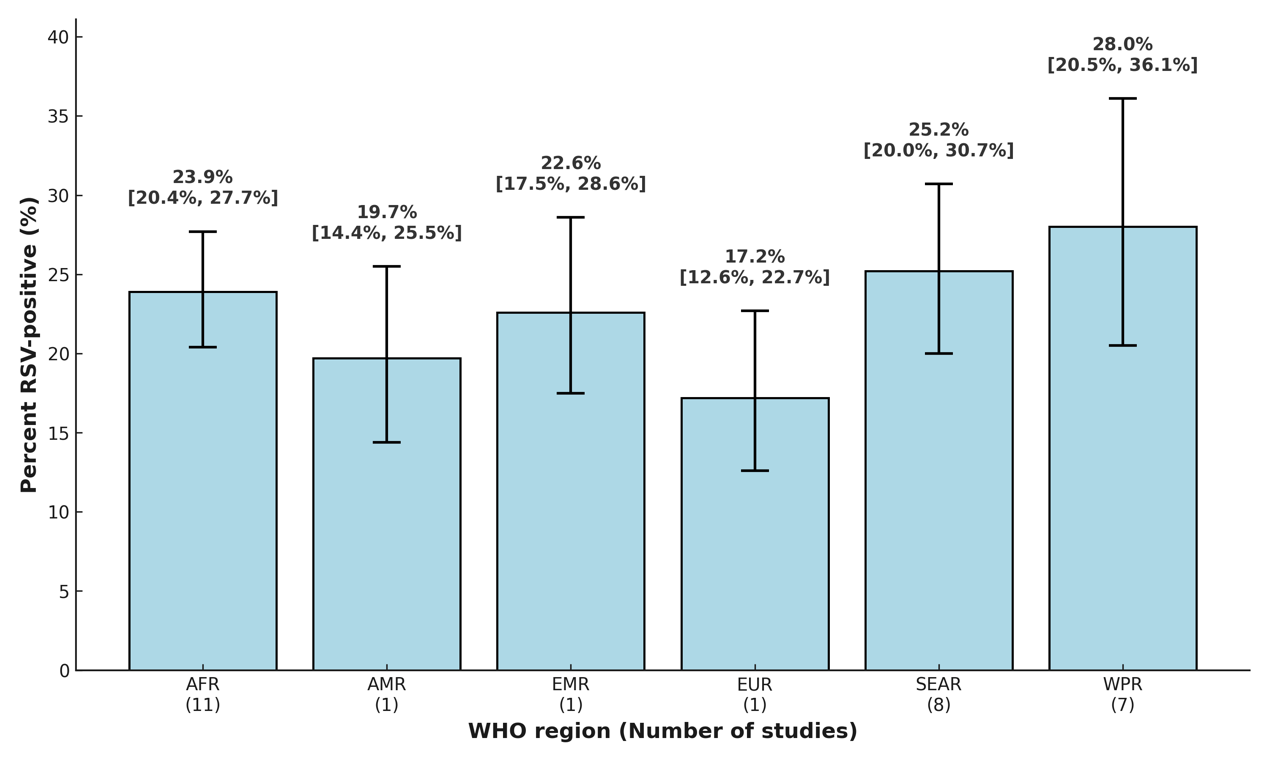


# Appendix 9. Global distribution of under five children RSV positive proportion eligible, peer-reviewed studies worldwide—based on data published from January 1, 2010 to October 14, 2022


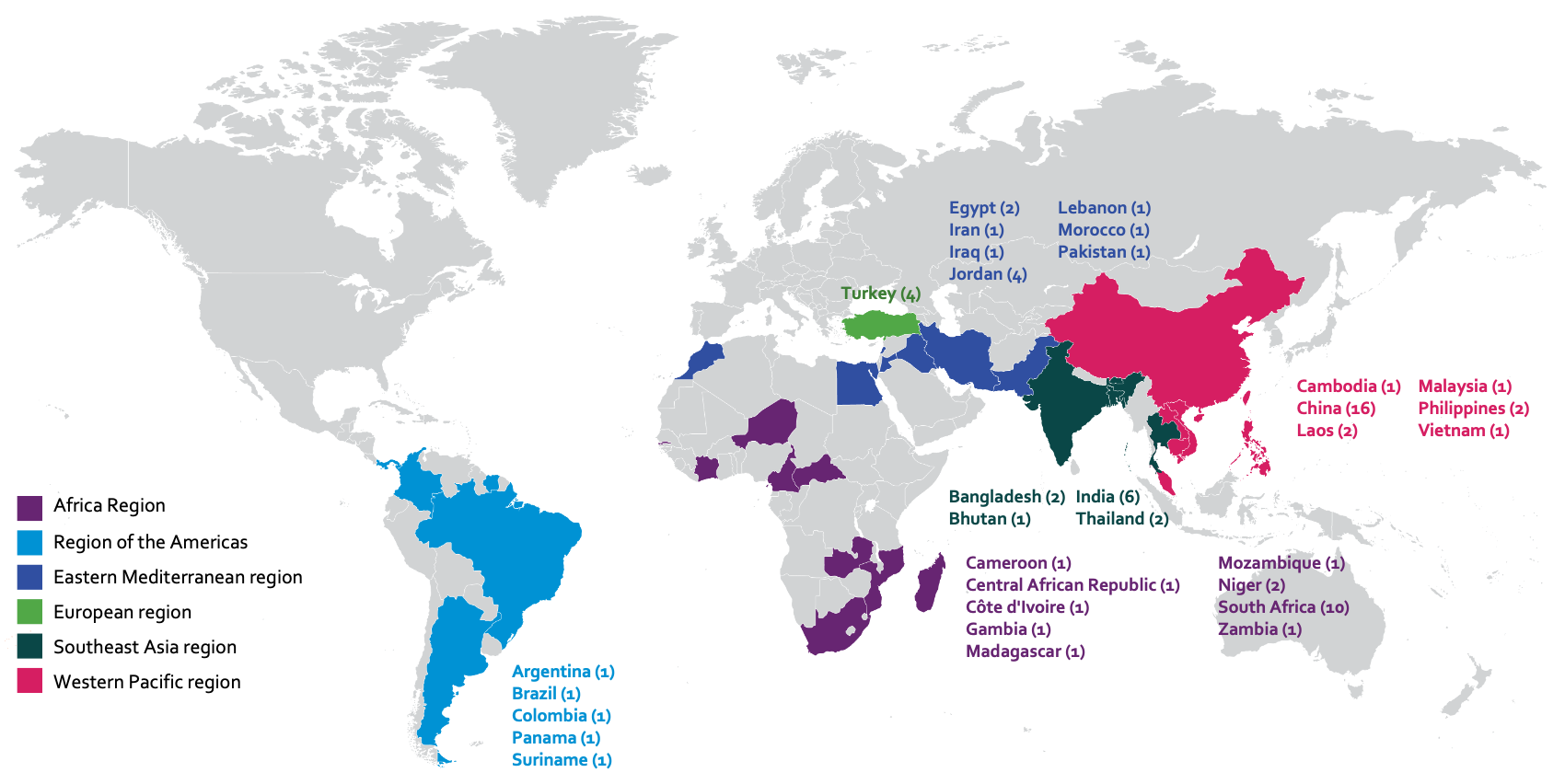


**References**

1. Page MJ, McKenzie JE, Bossuyt PM, et al. The PRISMA 2020 statement: an updated guideline for reporting systematic reviews. *Bmj* 2021; **372**: n71.

2. Bashir U, Nisar N, Arshad Y, et al. Respiratory syncytial virus and influenza are the key viral pathogens in children <2 years hospitalized with bronchiolitis and pneumonia in Islamabad Pakistan. *Arch Virol* 2017; **162**(3): 763-73.

3. Bénet T, Sánchez Picot V, Messaoudi M, et al. Microorganisms Associated With Pneumonia in Children <5 Years of Age in Developing and Emerging Countries: The GABRIEL Pneumonia Multicenter, Prospective, Case-Control Study. *Clin Infect Dis* 2017; **65**(4): 604-12.

4. Bhuyan GS, Hossain MA, Sarker SK, et al. Bacterial and viral pathogen spectra of acute respiratory infections in under-5 children in hospital settings in Dhaka city. *PLoS One* 2017; **12**(3): e0174488.

5. Biswal B, Dwibedi B, Hansa J, Kar S. Bacterial and Viral Pathogen Spectra of ARI among the Children Below 5 Years Age Group in Tribal and Coastal Regions of Odisha. *Indian Journal of Public Health Research and Development* 2018; **9**.

6. Bunthi C, Baggett HC, Gregory CJ, et al. Enhanced surveillance for severe pneumonia, Thailand 2010-2015. *BMC Public Health* 2019; **19**(Suppl 3): 472.

7. Chu FL, Li C, Chen L, Dong B, Qiu Y, Liu Y. Respiratory viruses among pediatric inpatients with acute lower respiratory tract infections in Jinan, China, 2016-2019. *J Med Virol* 2022; **94**(9): 4319-28.

8. Cohen C, Moyes J, Tempia S, et al. Epidemiology of Acute Lower Respiratory Tract Infection in HIV-Exposed Uninfected Infants. *Pediatrics* 2016; **137**(4).

9. Duyu M, Karakaya Z. VIRAL ETIOLOGY AND OUTCOME OF SEVERE LOWER RESPIRATORY TRACT INFECTIONS AMONG CRITICALLY ILL CHILDREN ADMITTED TO THE PICU. *Med Intensiva (Engl Ed)* 2020.

10. Fadugba OO, Haddadin Z, Muhimpundu S, et al. Respiratory Viruses Associated With Acute Wheezing in Hospitalized Young Children in Jordan. *J Pediatric Infect Dis Soc* 2021; **10**(4): 525-8.

11. Fan R, Fan C, Zhang J, et al. Respiratory syncytial virus subtype ON1/NA1/BA9 predominates in hospitalized children with lower respiratory tract infections. *J Med Virol* 2017; **89**(2): 213-21.

12. Finianos M, Issa R, Curran MD, et al. Etiology, seasonality, and clinical characterization of viral respiratory infections among hospitalized children in Beirut, Lebanon. *J Med Virol* 2016; **88**(11): 1874-81.

13. Geoghegan S, Erviti A, Caballero MT, et al. Mortality due to Respiratory Syncytial Virus. Burden and Risk Factors. *Am J Respir Crit Care Med* 2017; **195**(1): 96-103.

14. Gong C, Zhang T, Luo M, et al. Distribution of the atypical pathogens of community-acquired pneumonia to disease severity. *J Thorac Dis* 2018; **10**(11): 5991-6001.

15. Gurgel RQ, Bezerra PGM, Duarte M, et al. Relative frequency, Possible Risk Factors, Viral Codetection Rates, and Seasonality of Respiratory Syncytial Virus Among Children With Lower Respiratory Tract Infection in Northeastern Brazil. *Medicine (Baltimore)* 2016; **95**(15): e3090.

16. Halasa N, Williams J, Faouri S, et al. Natural history and epidemiology of respiratory syncytial virus infection in the Middle East: Hospital surveillance for children under age two in Jordan. *Vaccine* 2015; **33**(47): 6479-87.

17. Lamrani Hanchi A, Guennouni M, Rachidi M, et al. Epidemiology of Respiratory Pathogens in Children with Severe Acute Respiratory Infection and Impact of the Multiplex PCR Film Array Respiratory Panel: A 2-Year Study. *Int J Microbiol* 2021; **2021**: 2276261.

18. Hassan DA, Rachid SK, Ziebuhr J. A Single-Center Study of Viral Respiratory Tract Infections in Hospitalized Children From the Kurdistan Region of Iraq. *Glob Pediatr Health* 2018; **5**: 2333794x18784996.

19. Hatem A, Mohamed S, Abu Elhassan UE, et al. Clinical characteristics and outcomes of patients with severe acute respiratory infections (SARI): results from the Egyptian surveillance study 2010-2014. *Multidiscip Respir Med* 2019; **14**: 11.

20. Hatem AM, Abuelhassan UE, Mohamed SAA, Rizk MS, El-kholy A, Al-Harras M. Viral and atypical bacterial etiologies of severe acute respiratory infection (SARI) in Egyptian patients: epidemiological patterns and results from the sentinel surveillance study 2010–2014. *The Egyptian Journal of Chest Diseases and Tuberculosis* 2019; **68**(1).

21. Homaira N, Luby SP, Hossain K, et al. Respiratory Viruses Associated Hospitalization among Children Aged <5 Years in Bangladesh: 2010-2014. *PLoS One* 2016; **11**(2): e0147982.

22. Juliana AE, Tang MJ, Kemps L, et al. Viral causes of severe acute respiratory infection in hospitalized children and association with outcomes: A two-year prospective surveillance study in Suriname. *PLoS One* 2021; **16**(2): e0247000.

23. Jullien S, Pradhan D, Tshering T, et al. Pneumonia in children admitted to the national referral hospital in Bhutan: A prospective cohort study. *Int J Infect Dis* 2020; **95**: 74-83.

24. Kadjo HA, Adjogoua E, Dia N, et al. DETECTION OF NON-INFLUENZA VIRUSES IN ACUTE RESPIRATORY INFECTIONS IN CHILDREN UNDER FIVE-YEAR-OLD IN COTE D'IVOIRE (JANUARY - DECEMBER 2013). *Afr J Infect Dis* 2018; **12**(2): 78-88.

25. Kamigaki T, Aldey PP, Mercado ES, et al. Estimates of influenza and respiratory syncytial virus incidences with fraction modeling approach in Baguio City, the Philippines, 2012-2014. *Influenza Other Respir Viruses* 2017; **11**(4): 311-8.

26. Karabay. M. Investigation of the effect of the COVID-19 pandemic on viral respiratory tract infections in the neonatal intensive care unit. *FLORA* 2022; **27**(1): 151-7.

27. Kenmoe S, Tchendjou P, Vernet MA, et al. Viral etiology of severe acute respiratory infections in hospitalized children in Cameroon, 2011-2013. *Influenza Other Respir Viruses* 2016; **10**(5): 386-93.

28. Khuri-Bulos N, Lawrence L, Piya B, et al. Severe outcomes associated with respiratory viruses in newborns and infants: a prospective viral surveillance study in Jordan. *BMJ Open* 2018; **8**(5): e021898.

29. Klink T, Rankin DA, Piya B, et al. Evaluating the diagnostic accuracy of the WHO Severe Acute Respiratory Infection (SARI) criteria in Middle Eastern children under two years over three respiratory seasons. *PLoS One* 2020; **15**(4): e0232188.

30. Komoyo GF, Yambiyo BM, Manirakiza A, et al. Epidemiology and genetic characterization of respiratory syncytial virus in children with acute respiratory infections: Findings from the influenza sentinel surveillance network in Central African Republic, 2015 to 2018. *Health Sci Rep* 2021; **4**(2): e298.

31. Koul PA, Saha S, Kaul KA, et al. Respiratory syncytial virus among children hospitalized with severe acute respiratory infection in Kashmir, a temperate region in northern India. *J Glob Health* 2022; **12**: 04050.

32. Lagare A, Maïnassara HB, Issaka B, Sidiki A, Tempia S. Viral and bacterial etiology of severe acute respiratory illness among children < 5 years of age without influenza in Niger. *BMC Infect Dis* 2015; **15**: 515.

33. Lagare A, Ousmane S, Dano ID, et al. Molecular detection of respiratory pathogens among children aged younger than 5 years hospitalized with febrile acute respiratory infections: A prospective hospital-based observational study in Niamey, Niger. *Health Sci Rep* 2019; **2**(11): e137.

34. Li YT, Liang Y, Ling YS, Duan MQ, Pan L, Chen ZG. The spectrum of viral pathogens in children with severe acute lower respiratory tract infection: A 3-year prospective study in the pediatric intensive care unit. *J Med Virol* 2019; **91**(9): 1633-42.

35. Lin Y, Fu Y, Xu M, et al. Evaluation of a PCR/ESI-MS platform to identify respiratory viruses from nasopharyngeal aspirates. *J Med Virol* 2015; **87**(11): 1867-71.

36. Liu T, Li Z, Zhang S, et al. Viral Etiology of acute respiratory tract infections in hospitalized children and adults in Shandong Province, China. *Virol J* 2015; **12**: 168.

37. Luo HJ, Huang XB, Zhong HL, et al. Epidemiological characteristics and phylogenic analysis of human respiratory syncytial virus in patients with respiratory infections during 2011-2016 in southern China. *Int J Infect Dis* 2020; **90**: 5-17.

38. Mazumdar J, Chawla-Sarkar M, Rajendran K, et al. Burden of respiratory tract infections among paediatric in and out-patient units during 2010-11. *Eur Rev Med Pharmacol Sci* 2013; **17**(6): 802-8.

39. McMorrow ML, Tempia S, Walaza S, et al. The Impact of Human Immunodeficiency Virus Exposure on Respiratory Syncytial Virus-associated Severe Respiratory Illness in South African Infants, 2011-2016. *Clin Infect Dis* 2019; **69**(12): 2208-11.

40. McMorrow ML, Tempia S, Walaza S, et al. The Role of Human Immunodeficiency Virus in Influenza- and Respiratory Syncytial Virus-associated Hospitalizations in South African Children, 2011-2016. *Clin Infect Dis* 2019; **68**(5): 773-80.

41. Moleleki M, du Plessis M, Ndlangisa K, et al. Pathogens detected using a syndromic molecular diagnostic platform in patients hospitalized with severe respiratory illness in South Africa in 2017. *Int J Infect Dis* 2022; **122**: 389-97.

42. Moyes J, Cohen C, Pretorius M, et al. Epidemiology of respiratory syncytial virus-associated acute lower respiratory tract infection hospitalizations among HIV-infected and HIV-uninfected South African children, 2010-2011. *J Infect Dis* 2013; **208 Suppl 3**: S217-26.

43. Nguyen VH, Dubot-Pérès A, Russell FM, et al. Acute respiratory infections in hospitalized children in Vientiane, Lao PDR - the importance of Respiratory Syncytial Virus. *Sci Rep* 2017; **7**(1): 9318.

44. Nguyen VH, Russell FM, Dance DA, et al. Nasal or throat sampling is adequate for the detection of the human respiratory syncytial virus in children with acute respiratory infections. *J Med Virol* 2019; **91**(9): 1602-7.

45. Núñez-Samudio V, Landires I. Epidemiology of viral respiratory infections in a pediatric reference hospital in Central Panama. *BMC Infect Dis* 2021; **21**(1): 43.

46. Causes of severe pneumonia requiring hospital admission in children without HIV infection from Africa and Asia: the PERCH multi-country case-control study. *Lancet* 2019; **394**(10200): 757-79.

47. Okulu E, Akduman H, Tunç G, et al. The Epidemiological and Clinical Features of Neonates Hospitalized with Lower Respiratory Tract Viral Infections. *Turkish Journal of Pediatric Disease* 2017.

48. Pale M, Nacoto A, Tivane A, et al. Respiratory syncytial and influenza viruses in children under 2 years old with severe acute respiratory infection (SARI) in Maputo, 2015. *PLoS One* 2017; **12**(11): e0186735.

49. Parsania M, Poopak B, Pouriayevali MH, Haghighi S, Amirkhani A, Nateghian A. Detection of Human Metapneumovirus and Respiratory Syncytial Virus by Real-Time Polymerase Chain Reaction Among Hospitalized Young Children in Iran. *Jundishapur J Microbiol* 2016; **9**(3): e32974.

50. Pourakbari B, Mahmoudi S, Movahedi Z, et al. Viral etiology of acute lower respiratory tract infections in hospitalized young children in a children's referral hospital in Iran. *Turk J Pediatr* 2014; **56**(4): 354-9.

51. Pratheepamornkull T, Ratanakorn W, Samransamruajkit R, Poovorawan Y. CAUSATIVE AGENTS OF SEVERE COMMUNITY ACQUIRED VIRAL PNEUMONIA AMONG CHILDREN IN EASTERN THAILAND. *Southeast Asian J Trop Med Public Health* 2015; **46**(4): 650-6.

52. Pretorius MA, Tempia S, Walaza S, et al. The role of influenza, RSV and other common respiratory viruses in severe acute respiratory infections and influenza-like illness in a population with a high HIV sero-prevalence, South Africa 2012-2015. *J Clin Virol* 2016; **75**: 21-6.

53. Rabarison JH, Tempia S, Harimanana A, et al. Burden and epidemiology of influenza- and respiratory syncytial virus-associated severe acute respiratory illness hospitalization in Madagascar, 2011-2016. *Influenza Other Respir Viruses* 2019; **13**(2): 138-47.

54. Apoorva T Raju RD, Nikki Rai, Ajay Kumar, Rajni Gaind. A Prospective Study on Respiratory Viral Pathogens Causing Acute Lower Respiratory Tract Infections in Children below Five Years of Age at a Tertiary Care Hospital of India. *Journal of Clinical and Diagnostic Research* 2021; **15**(5): DC12-DC6.

55. Ren KY, Ren L, Deng Y, et al. [Epidemiological characteristics of respiratory syncytial virus in hospitalized children with acute lower respiratory tract infection in Chongqing, China, from 2013 to 2018: an analysis of 2 066 cases]. *Zhongguo Dang Dai Er Ke Za Zhi* 2021; **23**(1): 67-73.

56. Rueda ZV, Aguilar Y, Maya MA, et al. Etiology and the challenge of diagnostic testing of community-acquired pneumonia in children and adolescents. *BMC Pediatr* 2022; **22**(1): 169.

57. Simusika P, Bateman AC, Theo A, et al. Identification of viral and bacterial pathogens from hospitalized children with severe acute respiratory illness in Lusaka, Zambia, 2011-2012: a cross-sectional study. *BMC Infect Dis* 2015; **15**: 52.

58. Singh AK, Jain A, Jain B, et al. Viral aetiology of acute lower respiratory tract illness in hospitalised paediatric patients of a tertiary hospital: one year prospective study. *Indian J Med Microbiol* 2014; **32**(1): 13-8.

59. Subramoney K, Hellferscee O, Pretorius M, et al. Human bocavirus, coronavirus, and polyomavirus detected among patients hospitalised with severe acute respiratory illness in South Africa, 2012 to 2013. *Health Sci Rep* 2018; **1**(8): e59.

60. Swamy MA, Malhotra B, Janardhan Reddy PV, Tiwari J. Profile of respiratory pathogens causing acute respiratory infections in hospitalised children at Rajasthan a 4 year's study. *Indian J Med Microbiol* 2018; **36**(2): 163-71.

61. Tallo VL, Kamigaki T, Tan AG, et al. Estimating influenza outpatients' and inpatients' incidences from 2009 to 2011 in a tropical urban setting in the Philippines. *Influenza Other Respir Viruses* 2014; **8**(2): 159-68.

62. Tempia S, Moyes J, Cohen AL, et al. The national burden of influenza-like illness and severe respiratory illness overall and associated with nine respiratory viruses in South Africa, 2013-2015. *Influenza Other Respir Viruses* 2022; **16**(3): 438-51.

63. Toh TH, Hii KC, Fieldhouse JK, et al. High Prevalence of Viral Infections Among Hospitalized Pneumonia Patients in Equatorial Sarawak, Malaysia. *Open Forum Infect Dis* 2019; **6**(3): ofz074.

64. Valley-Omar Z, Tempia S, Hellferscee O, et al. Human respiratory syncytial virus diversity and epidemiology among patients hospitalized with severe respiratory illness in South Africa, 2012-2015. *Influenza Other Respir Viruses* 2022; **16**(2): 222-35.

65. Wen S, Lv F, Chen X, et al. Application of a nucleic acid-based multiplex kit to identify viral and atypical bacterial aetiology of lower respiratory tract infection in hospitalized children. *J Med Microbiol* 2019; **68**(8): 1211-8.

66. Xu L, He X, Zhang DM, et al. Surveillance and genome analysis of human bocavirus in patients with respiratory infection in Guangzhou, China. *PLoS One* 2012; **7**(9): e44876.

67. Xu W, Guo L, Dong X, et al. Detection of Viruses and Mycoplasma pneumoniae in Hospitalized Patients with Severe Acute Respiratory Infection in Northern China, 2015-2016. *Jpn J Infect Dis* 2018; **71**(2): 134-9.

68. Yan XL, Li YN, Tang YJ, et al. Clinical characteristics and viral load of respiratory syncytial virus and human metapneumovirus in children hospitaled for acute lower respiratory tract infection. *J Med Virol* 2017; **89**(4): 589-97.

69. Peng Y, Shu C, Fu Z, Li QB, Liu Z, Yan L. [Pathogen detection of 1 613 cases of hospitalized children with community acquired pneumonia]. *Zhongguo Dang Dai Er Ke Za Zhi* 2015; **17**(11): 1193-9.

70. Yoshihara K, Le MN, Okamoto M, et al. Association of RSV-A ON1 genotype with Increased Pediatric Acute Lower Respiratory Tract Infection in Vietnam. *Sci Rep* 2016; **6**: 27856.

71. Yu J, Xie Z, Zhang T, et al. Comparison of the prevalence of respiratory viruses in patients with acute respiratory infections at different hospital settings in North China, 2012-2015. *BMC Infect Dis* 2018; **18**(1): 72.

72. Yu J, Liu C, Xiao Y, et al. Respiratory Syncytial Virus Seasonality, Beijing, China, 2007-2015. *Emerg Infect Dis* 2019; **25**(6): 1127-35.

73. Yuan XH, Li YM, Shen YY, Yang J, Jin Y. Clinical and Th1/Th2 immune response features of hospitalized children with human rhinovirus infection. *J Med Virol* 2020; **92**(1): 26-33.

74. Zar HJ, Nduru P, Stadler JAM, et al. Early-life respiratory syncytial virus lower respiratory tract infection in a South African birth cohort: epidemiology and effect on lung health. *Lancet Glob Health* 2020; **8**(10): e1316-e25.
